# Genetic Alterations That Do or Do Not Occur Naturally; Consequences for Genome Edited Organisms in the Context of Regulatory Oversight

**DOI:** 10.3389/fbioe.2018.00213

**Published:** 2019-01-16

**Authors:** René Custers, Josep M. Casacuberta, Dennis Eriksson, László Sági, Joachim Schiemann

**Affiliations:** ^1^VIB, Ghent, Belgium; ^2^Centre for Research in Agricultural Genomics (CRAG), Barcelona, Spain; ^3^Department of Plant Breeding, Faculty of Landscape Architecture, Horticulture and Crop Production Science, Swedish University of Agricultural Sciences, Alnarp, Sweden; ^4^Centre for Agriculture Research, Hungarian Academy of Sciences (MTA), Martonvásár, Hungary; ^5^Federal Research Centre for Cultivated Plants, Julius Kühn-Institut, Quedlinburg, Germany

**Keywords:** genome editing, regulatory oversight, natural genetic alterations, GMO, classification, future policy

## Abstract

The ability to successfully exploit genome edited organisms for the benefit of food security and the environment will essentially be determined by the extent to which these organisms fall under specific regulatory provisions. In many jurisdictions the answer to this question is considered to depend on the genetic characteristics of the edited organism, and whether the changes introduced in its genome do (or do not) occur naturally. We provide here a number of key considerations to assist with this evaluation as well as a guide of concrete examples of genetic alterations with an assessment of their natural occurrence. These examples support the conclusion that for many of the common types of alterations introduced by means of genome editing, the resulting organisms would not be subject to specific biosafety regulatory provisions whenever novelty of the genetic combination is a crucial determinant.

## Introduction

The advances presented by genome editing including oligonucleotide-directed mutagenesis (ODM) and site-directed nuclease (SDN) technology have been widely recognized as a true revolution in our abilities to alter and improve genomes. One of the fields where these techniques are predicted to have a significant impact is plant breeding. Humans have selected genotypes more adapted to their needs and have improved agricultural practices ever since the early Neolithic period. The use of SDNs, and in particular CRISPR/Cas technology, will allow the introduction of additional genomic alterations efficiently and with an unprecedented level of precision. This technological innovation also presents regulatory challenges and leads to a number of questions. First, are such genome edited organisms subject to specific regulatory provisions related to biosafety? In many jurisdictions around the world, there is still legal uncertainty about this. And second, if the answer to the first question is no, should genome edited organisms nevertheless be subject to regulatory oversight that is stricter in any aspect than those which apply to conventionally bred organisms? The impact these techniques will have on plant breeding will greatly depend on how we answer these questions. To unlock the promise and potential of genome editing and to take responsibility for its development to benefit society there is an urgent need for a legal clarification based on correct scientific understanding. To support the decision-making process we aim to describe what types of genetic alterations do and do not occur naturally and estimate to what extent various alterations along a range from small to large genetic changes may occur in nature. Genome editing also has a lot of potential for the introduction of epigenetic changes. The scope of this paper, however, is limited to alterations in the primary sequence of the genetic material.

## Genomes in Evolution

Genomes have evolved over millions of years starting from the first development of living organisms, leading to the evolution of a wide variety of species. Indeed, naturally occurring mutations together with natural selection are the key factors driving evolution. Many organisms have had the capacity to adjust to specific environmental conditions and the plasticity of genomes has been one of the factors contributing to the ability to survive changing conditions. Over the years our understanding of the mechanisms underlying this evolution has grown significantly. On top of that, modern genome sequencing technology has revealed to us what type of alterations have occurred during evolution, domestication and breeding. If we want to determine what types of combinations of genetic material should be considered novel and are beyond what can “occur naturally by mating and/or natural recombination”—the phrase used in the EU GMO definition—it is important to have a closer look at these alterations.

Genetic information has to be faithfully transmitted during each cell division to allow the correct development and functioning of each organism. It also needs to be faithfully transmitted to the offspring in order to maintain species boundaries and biodiversity. However, some level of change needs to be generated to endow genetic information with the plasticity required for organisms and species adaptation to a changing environment. For this reason, most organisms have evolved mechanisms to ensure a high but imperfect fidelity in DNA replication, causing spontaneous mutations at a low rate, and equally efficient and imperfect mechanisms for repairing the DNA when damaged by endogenous or environmental mutagenic agents such as UV or radiation (Kunkel and Erie, 2015). This leads to a certain amount of natural mutations continuously being introduced into the genomes of all organisms. In the annual model plant *Arabidopsis thaliana*, with a genome size that is about 24 times smaller than the human genome, this mutation rate has been estimated to be 7 × 10^−9^ base substitutions per site per generation (Ossowski et al., 2010), which is approximately one substitution per genome per generation. In addition to these continuously arising mutations, genomes are equipped with repetitive and mobile genetic elements that promote additional mutations and genome rearrangements, which are much more discontinuous during evolution (Lisch, 2013). Finally, genomes are not completely isolated within the species boundaries and different species can exchange genetic information in nature through horizontal transfer, as it has been shown for rice to millet (Diao et al., 2006) or for the *Poa* to *Festuca* grass genera (Vallenback et al., 2008), or even between kingdoms such as from *Agrobacterium* to sweet potato (Kyndt et al., 2015). Moreover, the combination of two complete genomes through interspecific crosses, has also frequently occurred during plant genome evolution (Wendel, 2015).

All these fine or crude genetic changes are the raw material on which selection operates allowing species adaptation and evolution, both in the wild and under human direction. A look at the mutations that are at the origin of new characters selected during crop domestication and breeding shows that they cover a wide range of mutation types and mechanisms (Olsen and Wendel, 2013). These include point mutations causing amino acid changes, premature translation terminations and changes in transcript splicing or gene regulation. They also include transposon insertions and large deletions causing changes in coding or regulatory sequences in genes involved in plant and inflorescence architecture, seed shattering and dormancy, grain size and color, among many other characters that have been subject to farmer-driven selection (Olsen and Wendel, 2013). The domestication and genetic improvement of crops to serve human needs has also necessitated the incorporation of new genes from other species or even the combination of two complete genomes from different species. In fact, the domestication of most crops is the result of the combination of many different types of mutagenic events (see for instance Table 1 in Olsen and Wendel, 2013). As an example, bread wheat domestication required many independent mutations, including those at the two genes controlling seed shattering in the wild emmer wheat (Avni et al., 2017), different introgressions from wild related species, a whole genome duplication event in emmer wheat, and an interspecific cross between the domesticated emmer wheat and the wild goatgrass (*Aegilops tauschii*) (Gornicki and Faris, 2014). In general, crop domestication has required a significant number of mutations and genome rearrangements accumulated in genomes, and this has also been true during the whole history of crop breeding.

Whereas, all these individual mutations happened in nature spontaneously, they were artificially selected and combined by humans, who ultimately have made possible the large phenotypic diversity of today's crops and the radical differences crops present when compared to wild plants. One may even argue that the specific combinations of enhanced traits in all our crops are something that never would have occurred nor maintained without human intervention.

Plant breeding has been widely influenced by scientific progress during modern history, which has allowed expanding the range of techniques incorporated and boosted its sophistication. One of the key factors during this process was the increase in genetic variation available for breeding which was achieved both by expanding the gene pool that could be used for breeding and by enlarging the variability within the species by mutagenesis. The expansion of the gene pool was obtained by forcing crosses with increasingly distant species with the help, among others, of *in vitro* culture techniques allowing the rescue of the offspring of otherwise sterile crosses. Embryo rescue plays an important role in plant breeding programmes and is expected to retain or even broaden its significance, since plants obtained by embryo rescue do not have to be considered as genetically modified (Winkelmann et al., 2010).

The increase in variability within species through mutagenesis has been particularly successful and during the past 70 years more than 3,200 new crop varieties have been developed through mutation programs (cf. IAEA/FAO mutant variety database, https://mvd.iaea.org/), predominantly using γ-ray irradiation, but also other physical or chemical mutagens (Jankowicz-Cieslak and Till, 2015).

It is clear that during evolution, domestication and plant breeding a wide variety of genetic alterations have occurred and are still being introduced and further exploited. But not every type of alteration does or is likely to occur naturally. Alterations that cannot occur naturally are considered novel. It is for instance highly unlikely that organisms that are unrelated at any higher taxonomic level exchange large amounts of genetic material, although the examples given above show that horizontal gene transfer (HGT) is known to occur also among sexually incompatible eukaryotic/plant species.

## The Novelty Criterion in GMO Regulatory Regimes

The Cartagena Protocol on Biosafety to the Convention on Biological Diversity serves as a framework promoting international harmonization in the legislation of GMOs (Cartagena Protocol, 2000). This Protocol does not use the term GMO but defines a “living modified organism” (LMO) as “any living organism that possesses a novel combination of genetic material developed through modern biotechnology.” In the Cartagena Protocol, the mere use of a technique of modern biotechnology is not enough to trigger regulatory oversight. The resulting organism additionally needs to possess a novel combination of genetic material. The combination of genetic material needs to be beyond what can occur naturally by mating and/or natural recombination. We deliberately use these latter words because also the EU GMO definition uses a similar phrasing. But what does “beyond what can occur naturally by mating and/or natural recombination” actually mean? As a next step we will therefore go over a list of concrete examples of genetic alterations in different species and determine, based on our current understanding of biology, whether these alterations do occur naturally.

## Alterations Beyond What Can Occur Naturally

In Table 1 we provide 15 concrete examples of genetic alterations. We do not specify by what means these alterations have been introduced, but in line with the Cartagena Protocol definitions they would only result in the formation of a specifically regulated organism, if—besides having resulted in the formation of a genetic combination that does not occur naturally by mating and/or natural recombination—they also have been achieved by a method that does not occur naturally. We propose that the wording “does not occur” should be interpreted such that the alterations are extremely unlikely to occur. Those that are more likely to occur in nature or as a result of human intervention via conventional breeding approaches would not be considered to result in the formation of a specifically regulated organism.

**Table 1:** Genetic alterations and the proposed classification of organisms in the context of GMO regulatory oversight.

	**Organism**	**Description of the alteration**	**Does it occur in nature?**	**Likeliness of occurrence**	Alteration beyond what does occur naturally by mating and/or natural recombination^*^	**Motivation**
1	Any plant	Single point mutation	YES	Very likely	NO	The mutation rate has been estimated in different plants to be on average 1 mutation per genome per generation, meaning that every single plant will contain, at least, one new mutation. See also the Appendix in supplementary material.
2	Any plant	2,000 point mutations	Perhaps only in a nuclear fall-out	Extremely unlikely	YES	This is the example of classical mutagenesis using a radioactive source to induce random mutations. The organisms resulting from this intervention are exempted from the scope of the EU GMO directive on the condition that no recombinant nucleic acid molecules or GMOs have been used.
3	Any plant	Short deletions (a few bp)	YES	Very likely	NO	Such one base pair deletions occur on a regular basis. Mostly after the occurrence of a double-strand break.
4	Any plant	Larger deletions (a few kbp)	YES	It does happen	NO	Larger deletions also do occur, but with a lower frequency. In viruses such deletions occur rather frequently during processes of attenuation.
5	Any plant	Short insertions (a few bp)	YES	Very likely	NO	Small insertions also occur on a regular basis. Similar as with small deletions such insertions may occur, for example after a double-strand break.
6	Maize	50 kb exogenous DNA insertion	YES	Very unlikely	YES	Such size of insertions do occur in very rare occasions, but the larger the insertion the more unlikely they become.
7	Any plant	2 kb exogenous DNA insertion from a non-related source (a transgene)	YES	Very unlikely	YES	Even though there are rare events of horizontal gene transfer, it is very unlikely that such an event occurs. This is also what the legislation truly intended to have covered by the GMO legislation.
8	Any plant	A cisgene flanked by the T-DNA left and right borders	NO	Extremely unlikely	YES?	There are no known examples of such types of alterations occurring in nature. Where T-DNA has been introduced spontaneously, it was with sequences stemming from *A.tumefaciens* in between.
9	Any plant	An allele swap	YES	It does happen	NO	Allele swaps do occur, especially as a result of recombination during meiosis.
10	Any plant	Cisgene addition	YES	It does happen	NO/YES	The difference with an allele swap is that the cisgene may be present anywhere in the genome, not necessarily on its 'natural' location. It is known however that in plants genes on a regular basis get duplicated, resulting in an extra copy of the gene being inserted at another location in the genome, away from its original ‘natural' location.
11	Any plant	Intragene addition	?	Extremely unlikely	YES	The rearrangement of regulatory sequences does occur in nature and is actually important in evolutionary terms. But it is extremely unlikely to occur within a few generations in the context of plant breeding.
12	Plants that cannot naturally be infected by *A. tumefaciens*	T-DNA insertion	NO	Extremely unlikely	YES	When a plant cannot naturally be infected by *A. tumefaciens*, then the insertion of T-DNA cannot occur.
13	Plants that can be naturally infected by *A. tumefaciens* such as *Nicotiana* spp., sweet potato and *Linaria* spp	T-DNA insertion	YES	Unlikely, but it has happened	NO	*A. tumefaciens* can naturally infect a wide variety of plants, but this usually is a local infection resulting in the formation of crown galls, without the T-DNA being inserted into the reproductive DNA. However, this has happened during evolution of for example sweet potato.
14	Any plant	Genome duplication	YES	It does happen	NO	Genome duplications do spontaneously happen in nature but have even more been exploited by man to strengthen certain characteristics of crops.
15	Any plant	Transposon insertion	YES	It does happen	NO	Transposons mobile genetic elements that account for an important fraction of all plant genomes. Its movement generates mutations that are at the origin of many traits selected by humans to domesticate wild plants of breed new varieties.

* *As proposed in the article organisms should only be considered as GMO subject to specific regulatory oversight when they fulfill two criteria: (1) the alteration must have been achieved by means of a technical intervention that does not occur naturally, and (2) the genetic combination formed must be beyond what does occur naturally by mating and/or natural recombination*.

The first two examples in Table 1 are about point mutations of which we state that the likelihood of occurrence is very high. This can be easily substantiated by performing sequence analyses to estimate the occurrence of single nucleotide polymorphisms (SNPs) in genes in different crops. In most genes one will find dozens if not hundreds of SNPs. The more varieties of a crop are subject to the analysis, the more SNPs are generally found. In a separate appendix to this paper we provide such an analysis for the acetolactate synthase (ALS) gene in Arabidopsis, wheat and rice. This can be seen as an illustrative example for the occurrence of SNPs in any gene in a crop. Similarly, one can perform sequence analyses for determining the natural occurrence of the other types of genetic alterations that we describe in Table 1, as well.

## Emerging Conclusions and Principles

From what we have described above it is apparent that quite a lot of alterations are already occurring naturally. If we then go over the more concrete examples of genetic alterations (Table 1), in many occasions we get a rather clear picture. When such alterations are deliberately introduced by means of techniques that do not occur naturally, and the regulatory framework uses novelty of the genetic combination as a criterion, then the resulting organisms either would clearly classify as a specifically regulated organism or would clearly not classify as such. This is for instance the case for single point mutations or for deletions of any size, which in that context would not be considered to result in the formation of a specifically regulated organism, or for the introduction of a transgene, which does result in the formation of such an organism. The deliberate and simultaneous introduction of a very large amount of specific point mutations would under such legal approach also be considered to result in the formation of a specifically regulated organism, even though each individual mutation can occur naturally.

But there are also areas that are less clear. Exactly how many point mutations can be simultaneously introduced before the organism would become a specifically regulated organism? And how many sequential base pairs of exogenous or recombinant DNA, stemming from a non-crossable source, can be introduced before the organism becomes a specifically regulated organism? For sure, the more point mutations and the longer the stretch of exogenous DNA, the less likely it is that this would occur spontaneously in nature. Drawing a line will inevitably be arbitrary, however for regulatory purposes this will be necessary.

Concerning the number of point mutations one could perhaps debate why this is important to discuss in detail. Yet, it might still be relevant to determine how many point mutations are allowed to happen because it will largely determine whether the resulting organism would have to be classified as a specifically regulated organism or not. Are 10 simultaneous point mutations acceptable? For sure the introduction of 40 SNPs is acceptable if they are introduced by means of an allele swap, where one existing allele is replaced by another allele originating from the organisms' gene pool. Another question is whether the introduction of different mutations in consecutive rounds of intervention at a certain point would trigger the legislation. In other words: would the simultaneous introduction of 200 point mutations be considered to lead to the formation of a novel combination of genetic material and therefore become a specifically regulated organism, whereas the accumulated introduction of 200 single point mutations would not? These intricate but realistic questions need pragmatic answers.

## Recommendations

The scheme and the concrete examples that we have presented will help to clarify the regulatory status of genome edited organisms within different regulatory frameworks. Several competent authorities in the world have recently clarified the scope of their legislation. USDA-APHIS has stated that organisms with single point mutations, deletions of any size, and in which genes from compatible species have been introduced are not a regulated article under its biotechnology regulations. In their view such organisms do not require specific regulatory scrutiny because they could otherwise have been developed through traditional breeding techniques. Conventionally bred varieties are considered by the US regulators to present a risk level one should not be forced to go below. In Argentina and Brazil regulatory procedures have been introduced through which the regulatory bodies can determine on a case-by-case basis whether something would be regulated as a GMO (Whelan and Lema, 2015). They use the novelty criterion from the Cartagena Protocol LMO definition as their guiding principle. The outcomes of these procedures so far show that also they do not regard organisms with point mutations or (small) deletions, or any other that could have occurred through conventional breeding or by natural, spontaneous mutations, as GMOs that require specific scrutiny.

In the EU, the recent ruling of the Court of Justice of the European Union considers genome edited organisms as GMOs that do not fall under the existing exemption for organisms resulting from conventional mutagenesis (CJEU).^1^ The motivation of the ruling leaves very little room for a more product-oriented interpretation of the phrase “has been altered in a way that does not occur naturally by mating and or natural recombination” in the EU GMO definition. The Court has not used the novelty criterion, even though it could have. This implies that organisms that have edits that can or do occur naturally, will have to follow the same regulatory procedures as GMOs, including a detailed analysis of possible risks. This does not comply with the principles set out in the Cartagena Protocol on Biosafety and would be disproportionate and scientifically unsound. In the last few months, different proposals to solve this situation have been proposed. First, the existing EU GMO legal framework could be modified following for instance the proposal put forward by the Netherlands (Eriksson et al., 2018). This would allow including genome editing techniques in the list of techniques exempted from the regulation. Another option that has also been proposed would be to revert the trend of the last 15 years limiting the latitude of scientist and risk assessors applying the case-by-case approach to the risk analysis of GMOs as laid down in Directives 90/220/EEC and 2001/18/EC (Casacuberta and Puigdomènech, 2018). Whatever the path followed, the principles developed in this article should help to adjust the legal framework to each use of genome editing techniques and perform a more proportionate risk assessment of genome edited organisms.

It is important to note that being classified or not as an LMO under the Cartagena Protocol, a GMO under EU legislation, a regulated article under the USDA-APHIS plant pest legislative framework, is not *per se* a safety related issue. However, it is true that especially the EU GMO regulatory framework is foremost applied to organisms that contain novel genetic combinations beyond what does occur naturally by mating and/or natural recombination, and subject these to pre-market risk assessment under the pretext that these organisms may carry with them a risk due to the genetic novelty *per se*. Mutations, whether naturally occurring or man-made, such as limited nucleotide changes could also result in phenotypic changes presenting a hazard and the legislator has not seen a need to place them under additional scrutiny. This is because the regulator has considered the products of traditional breeding techniques including mutagenesis to present a risk level that is acceptable.

Breeders are not required by law to perform a pre-market risk assessment and get a market authorization for new varieties based on the use of conventional breeding techniques including conventional mutagenesis. But their products nevertheless need to be safe for human consumption and the environment. If they are not, other food and environmental legislation such as the US legislation on food safety, the EU general food law and the EU environmental liability legislation enters into force to correct them and, if necessary, hold the developer accountable (De Jong et al., 2018).

## Author Contributions

All authors have jointly contributed to the manuscript, with particular contribution of JC to the text on genomes in evolution and LS to the appendices.

### Conflict of Interest Statement

The authors declare that the research was conducted in the absence of any commercial or financial relationships that could be construed as a potential conflict of interest.

## Footnotes

^1^http://curia.europa.eu/juris/document/document.jsf?text=&docid=207002&pageIndex=0&doclang=EN&mode=req&dir=&occ=first&part=1&cid=6947981
