# Supplementary material for: Genetic Alterations That Do or Do Not Occur Naturally; Consequences for Genome Edited Organisms in the Context of Regulatory Oversight
Source: Front Bioeng Biotechnol. 2019 Jan 16;6:213. doi: 10.3389/fbioe.2018.00213 (PMC6343457; doi:10.3389/fbioe.2018.00213)
Supplement: Supplementary file 2 [file Table_2.docx]

Appendix Table S2: rice natural SNPs

Rice (japonica) ALS: Os02g0510200, chr. 2 18,236,120-18,238,054; CDS 1,935 bp, 644 AA

M A T T A A A A A A A L S A A A T A K T G R K N H Q R H H V L P A R G R V G A

**ATG**GCTACGACCGCC[G](http://plants.ensembl.org/Oryza_sativa/ZMenu/TextSequence?db=core;factorytype=Location;g=Os02g0510200;r=2:18236073-18238350;t=Os02t0510200-01;v=rs18703671;vf=4198713)CGGCCGC[G](http://plants.ensembl.org/Oryza_sativa/ZMenu/TextSequence?db=core;factorytype=Location;g=Os02g0510200;r=2:18236073-18238350;t=Os02t0510200-01;v=rs18703680;vf=4198714)GCCGCC[G](http://plants.ensembl.org/Oryza_sativa/ZMenu/TextSequence?db=core;factorytype=Location;g=Os02g0510200;r=2:18236073-18238350;t=Os02t0510200-01;v=vcZ269LAE;vf=4198715)CC[C](http://plants.ensembl.org/Oryza_sativa/ZMenu/TextSequence?db=core;factorytype=Location;g=Os02g0510200;r=2:18236073-18238350;t=Os02t0510200-01;v=vcZ269LAF;vf=4198716)TGTCCGCCGC[C](http://plants.ensembl.org/Oryza_sativa/ZMenu/TextSequence?db=core;factorytype=Location;g=Os02g0510200;r=2:18236073-18238350;t=Os02t0510200-01;v=rs18703689;vf=4198717)GCG[A](http://plants.ensembl.org/Oryza_sativa/ZMenu/TextSequence?db=core;factorytype=Location;g=Os02g0510200;r=2:18236073-18238350;t=Os02t0510200-01;v=rs18703698;vf=4198718)CGGCC[A](http://plants.ensembl.org/Oryza_sativa/ZMenu/TextSequence?db=core;factorytype=Location;g=Os02g0510200;r=2:18236073-18238350;t=Os02t0510200-01;v=rs18703707;vf=4198719)AGACCGGCCGTAAGAACCACCAGCGACACCACGTC[C](http://plants.ensembl.org/Oryza_sativa/ZMenu/TextSequence?db=core;factorytype=Location;g=Os02g0510200;r=2:18236073-18238350;t=Os02t0510200-01;v=vcZ269LAG;vf=4198720)TTCCCGCTCGAGGCCGGGTGGGGGC

A A V R C S A V S P V T P P S P A P P A T P L R P W G P A E P R K G A D I L

GGCGGCGGTCAGGTGCTCGGCGGTGTCCCCGGTCACCCCGCCGTCCCCGGCGCCGCCGGCCACGCCGCTCCGGCCGTGGGGG[C](http://plants.ensembl.org/Oryza_sativa/ZMenu/TextSequence?db=core;factorytype=Location;g=Os02g0510200;r=2:18236073-18238350;t=Os02t0510200-01;v=vcZ269LAH;vf=4198721)CGGCCGAGCCCCGCAAGGGCGCGGACATCCTCG

V E A L E R C G V S D V F A Y P G **G** **A** S M E I H Q A L T R S P V I T N H L F R

TGGAGGCGCT[G](http://plants.ensembl.org/Oryza_sativa/ZMenu/TextSequence?db=core;factorytype=Location;g=Os02g0510200;r=2:18236073-18238350;t=Os02t0510200-01;v=rs18703716;vf=4198722)[G](http://plants.ensembl.org/Oryza_sativa/ZMenu/TextSequence?db=core;factorytype=Location;g=Os02g0510200;r=2:18236073-18238350;t=Os02t0510200-01;v=rs18703725;vf=4198723)[A](http://plants.ensembl.org/Oryza_sativa/ZMenu/TextSequence?db=core;factorytype=Location;g=Os02g0510200;r=2:18236073-18238350;t=Os02t0510200-01;v=rs18703734;vf=4198724)GC[G](http://plants.ensembl.org/Oryza_sativa/ZMenu/TextSequence?db=core;factorytype=Location;g=Os02g0510200;r=2:18236073-18238350;t=Os02t0510200-01;v=rs18703743;vf=4198725)G[T](http://plants.ensembl.org/Oryza_sativa/ZMenu/TextSequence?db=core;factorytype=Location;g=Os02g0510200;r=2:18236073-18238350;t=Os02t0510200-01;v=rs18703752;vf=4198726)GCGG[C](http://plants.ensembl.org/Oryza_sativa/ZMenu/TextSequence?db=core;factorytype=Location;g=Os02g0510200;r=2:18236073-18238350;t=Os02t0510200-01;v=rs18703761;vf=4198727)GTCAGCG[A](http://plants.ensembl.org/Oryza_sativa/ZMenu/TextSequence?db=core;factorytype=Location;g=Os02g0510200;r=2:18236073-18238350;t=Os02t0510200-01;v=rs18703770;vf=4198728)CG[T](http://plants.ensembl.org/Oryza_sativa/ZMenu/TextSequence?db=core;factorytype=Location;g=Os02g0510200;r=2:18236073-18238350;t=Os02t0510200-01;v=rs18703779;vf=4198729)[G](http://plants.ensembl.org/Oryza_sativa/ZMenu/TextSequence?db=core;factorytype=Location;g=Os02g0510200;r=2:18236073-18238350;t=Os02t0510200-01;v=rs18703788;vf=4198730)[T](http://plants.ensembl.org/Oryza_sativa/ZMenu/TextSequence?db=core;factorytype=Location;g=Os02g0510200;r=2:18236073-18238350;t=Os02t0510200-01;v=rs18703797;vf=4198731)[T](http://plants.ensembl.org/Oryza_sativa/ZMenu/TextSequence?db=core;factorytype=Location;g=Os02g0510200;r=2:18236073-18238350;t=Os02t0510200-01;v=rs18703806;vf=4198732)C[G](http://plants.ensembl.org/Oryza_sativa/ZMenu/TextSequence?db=core;factorytype=Location;g=Os02g0510200;r=2:18236073-18238350;t=Os02t0510200-01;v=rs18703815;vf=4198733)CC[T](http://plants.ensembl.org/Oryza_sativa/ZMenu/TextSequence?db=core;factorytype=Location;g=Os02g0510200;r=2:18236073-18238350;t=Os02t0510200-01;v=rs18703824;vf=4198734)[A](http://plants.ensembl.org/Oryza_sativa/ZMenu/TextSequence?db=core;factorytype=Location;g=Os02g0510200;r=2:18236073-18238350;t=Os02t0510200-01;v=rs18703833;vf=4198735)CCC[G](http://plants.ensembl.org/Oryza_sativa/ZMenu/TextSequence?db=core;factorytype=Location;g=Os02g0510200;r=2:18236073-18238350;t=Os02t0510200-01;v=rs18703842;vf=4198736)GGC**GGC**[**G**](http://plants.ensembl.org/Oryza_sativa/ZMenu/TextSequence?db=core;factorytype=Location;g=Os02g0510200;r=2:18236073-18238350;t=Os02t0510200-01;v=rs18703851;vf=4198737)**C**[**G**](http://plants.ensembl.org/Oryza_sativa/ZMenu/TextSequence?db=core;factorytype=Location;g=Os02g0510200;r=2:18236073-18238350;t=Os02t0510200-01;v=rs18703860;vf=4198738)[T](http://plants.ensembl.org/Oryza_sativa/ZMenu/TextSequence?db=core;factorytype=Location;g=Os02g0510200;r=2:18236073-18238350;t=Os02t0510200-01;v=rs18703869;vf=4198739)[C](http://plants.ensembl.org/Oryza_sativa/ZMenu/TextSequence?db=core;factorytype=Location;g=Os02g0510200;r=2:18236073-18238350;t=Os02t0510200-01;v=rs18703878;vf=4198740)CAT[G](http://plants.ensembl.org/Oryza_sativa/ZMenu/TextSequence?db=core;factorytype=Location;g=Os02g0510200;r=2:18236073-18238350;t=Os02t0510200-01;v=rs18703887;vf=4198741)G[A](http://plants.ensembl.org/Oryza_sativa/ZMenu/TextSequence?db=core;factorytype=Location;g=Os02g0510200;r=2:18236073-18238350;t=Os02t0510200-01;v=rs18703896;vf=4198742)GATCC[A](http://plants.ensembl.org/Oryza_sativa/ZMenu/TextSequence?db=core;factorytype=Location;g=Os02g0510200;r=2:18236073-18238350;t=Os02t0510200-01;v=rs18703904;vf=4198743)C[C](http://plants.ensembl.org/Oryza_sativa/ZMenu/TextSequence?db=core;factorytype=Location;g=Os02g0510200;r=2:18236073-18238350;t=Os02t0510200-01;v=rs18703913;vf=4198744)[A](http://plants.ensembl.org/Oryza_sativa/ZMenu/TextSequence?db=core;factorytype=Location;g=Os02g0510200;r=2:18236073-18238350;t=Os02t0510200-01;v=rs18703922;vf=4198745)[G](http://plants.ensembl.org/Oryza_sativa/ZMenu/TextSequence?db=core;factorytype=Location;g=Os02g0510200;r=2:18236073-18238350;t=Os02t0510200-01;v=rs18703931;vf=4198746)GCGC[T](http://plants.ensembl.org/Oryza_sativa/ZMenu/TextSequence?db=core;factorytype=Location;g=Os02g0510200;r=2:18236073-18238350;t=Os02t0510200-01;v=rs18703940;vf=4198747)GACGCGCT[C](http://plants.ensembl.org/Oryza_sativa/ZMenu/TextSequence?db=core;factorytype=Location;g=Os02g0510200;r=2:18236073-18238350;t=Os02t0510200-01;v=rs18703949;vf=4198748)C[C](http://plants.ensembl.org/Oryza_sativa/ZMenu/TextSequence?db=core;factorytype=Location;g=Os02g0510200;r=2:18236073-18238350;t=Os02t0510200-01;v=rs18703958;vf=4198749)CG[G](http://plants.ensembl.org/Oryza_sativa/ZMenu/TextSequence?db=core;factorytype=Location;g=Os02g0510200;r=2:18236073-18238350;t=Os02t0510200-01;v=rs18703967;vf=4198750)TCATCACCAACCACCTCTTCCGC

H E Q G E A F A A S G Y A R A S G R V G V C V A T S G P G A T N L V S A L A D

CA[C](http://plants.ensembl.org/Oryza_sativa/ZMenu/TextSequence?db=core;factorytype=Location;g=Os02g0510200;r=2:18236073-18238350;t=Os02t0510200-01;v=rs18703976;vf=4198751)[G](http://plants.ensembl.org/Oryza_sativa/ZMenu/TextSequence?db=core;factorytype=Location;g=Os02g0510200;r=2:18236073-18238350;t=Os02t0510200-01;v=rs18703985;vf=4198752)AGCAGGGC[G](http://plants.ensembl.org/Oryza_sativa/ZMenu/TextSequence?db=core;factorytype=Location;g=Os02g0510200;r=2:18236073-18238350;t=Os02t0510200-01;v=rs18703994;vf=4198753)AGGC[G](http://plants.ensembl.org/Oryza_sativa/ZMenu/TextSequence?db=core;factorytype=Location;g=Os02g0510200;r=2:18236073-18238350;t=Os02t0510200-01;v=rs18704003;vf=4198754)[T](http://plants.ensembl.org/Oryza_sativa/ZMenu/TextSequence?db=core;factorytype=Location;g=Os02g0510200;r=2:18236073-18238350;t=Os02t0510200-01;v=rs18704012;vf=4198755)[T](http://plants.ensembl.org/Oryza_sativa/ZMenu/TextSequence?db=core;factorytype=Location;g=Os02g0510200;r=2:18236073-18238350;t=Os02t0510200-01;v=rs18704021;vf=4198756)CGC[G](http://plants.ensembl.org/Oryza_sativa/ZMenu/TextSequence?db=core;factorytype=Location;g=Os02g0510200;r=2:18236073-18238350;t=Os02t0510200-01;v=rs18704030;vf=4198757)GC[G](http://plants.ensembl.org/Oryza_sativa/ZMenu/TextSequence?db=core;factorytype=Location;g=Os02g0510200;r=2:18236073-18238350;t=Os02t0510200-01;v=rs18704039;vf=4198758)T[C](http://plants.ensembl.org/Oryza_sativa/ZMenu/TextSequence?db=core;factorytype=Location;g=Os02g0510200;r=2:18236073-18238350;t=Os02t0510200-01;v=rs18704048;vf=4198759)[C](http://plants.ensembl.org/Oryza_sativa/ZMenu/TextSequence?db=core;factorytype=Location;g=Os02g0510200;r=2:18236073-18238350;t=Os02t0510200-01;v=rs18704057;vf=4198760)[G](http://plants.ensembl.org/Oryza_sativa/ZMenu/TextSequence?db=core;factorytype=Location;g=Os02g0510200;r=2:18236073-18238350;t=Os02t0510200-01;v=rs18704066;vf=4198761)GG[T](http://plants.ensembl.org/Oryza_sativa/ZMenu/TextSequence?db=core;factorytype=Location;g=Os02g0510200;r=2:18236073-18238350;t=Os02t0510200-01;v=rs18704075;vf=4198762)ACGCGCGCGC[G](http://plants.ensembl.org/Oryza_sativa/ZMenu/TextSequence?db=core;factorytype=Location;g=Os02g0510200;r=2:18236073-18238350;t=Os02t0510200-01;v=rs18704084;vf=4198763)TCC[G](http://plants.ensembl.org/Oryza_sativa/ZMenu/TextSequence?db=core;factorytype=Location;g=Os02g0510200;r=2:18236073-18238350;t=Os02t0510200-01;v=rs18704093;vf=4198764)G[C](http://plants.ensembl.org/Oryza_sativa/ZMenu/TextSequence?db=core;factorytype=Location;g=Os02g0510200;r=2:18236073-18238350;t=Os02t0510200-01;v=rs18704102;vf=4198765)[C](http://plants.ensembl.org/Oryza_sativa/ZMenu/TextSequence?db=core;factorytype=Location;g=Os02g0510200;r=2:18236073-18238350;t=Os02t0510200-01;v=rs18704111;vf=4198766)GCG[T](http://plants.ensembl.org/Oryza_sativa/ZMenu/TextSequence?db=core;factorytype=Location;g=Os02g0510200;r=2:18236073-18238350;t=Os02t0510200-01;v=rs18704120;vf=4198767)C[G](http://plants.ensembl.org/Oryza_sativa/ZMenu/TextSequence?db=core;factorytype=Location;g=Os02g0510200;r=2:18236073-18238350;t=Os02t0510200-01;v=rs18704129;vf=4198768)[G](http://plants.ensembl.org/Oryza_sativa/ZMenu/TextSequence?db=core;factorytype=Location;g=Os02g0510200;r=2:18236073-18238350;t=Os02t0510200-01;v=rs18704138;vf=4198769)[G](http://plants.ensembl.org/Oryza_sativa/ZMenu/TextSequence?db=core;factorytype=Location;g=Os02g0510200;r=2:18236073-18238350;t=Os02t0510200-01;v=rs18704147;vf=4198770)GTCTG[C](http://plants.ensembl.org/Oryza_sativa/ZMenu/TextSequence?db=core;factorytype=Location;g=Os02g0510200;r=2:18236073-18238350;t=Os02t0510200-01;v=rs18704156;vf=4198771)GTC[G](http://plants.ensembl.org/Oryza_sativa/ZMenu/TextSequence?db=core;factorytype=Location;g=Os02g0510200;r=2:18236073-18238350;t=Os02t0510200-01;v=rs18704165;vf=4198772)CC[A](http://plants.ensembl.org/Oryza_sativa/ZMenu/TextSequence?db=core;factorytype=Location;g=Os02g0510200;r=2:18236073-18238350;t=Os02t0510200-01;v=rs18704174;vf=4198773)[C](http://plants.ensembl.org/Oryza_sativa/ZMenu/TextSequence?db=core;factorytype=Location;g=Os02g0510200;r=2:18236073-18238350;t=Os02t0510200-01;v=rs18704183;vf=4198774)CTCC[G](http://plants.ensembl.org/Oryza_sativa/ZMenu/TextSequence?db=core;factorytype=Location;g=Os02g0510200;r=2:18236073-18238350;t=Os02t0510200-01;v=rs18704192;vf=4198775)[G](http://plants.ensembl.org/Oryza_sativa/ZMenu/TextSequence?db=core;factorytype=Location;g=Os02g0510200;r=2:18236073-18238350;t=Os02t0510200-01;v=rs18704201;vf=4198776)C[C](http://plants.ensembl.org/Oryza_sativa/ZMenu/TextSequence?db=core;factorytype=Location;g=Os02g0510200;r=2:18236073-18238350;t=Os02t0510200-01;v=rs18704210;vf=4198777)CC[G](http://plants.ensembl.org/Oryza_sativa/ZMenu/TextSequence?db=core;factorytype=Location;g=Os02g0510200;r=2:18236073-18238350;t=Os02t0510200-01;v=rs18704219;vf=4198778)[G](http://plants.ensembl.org/Oryza_sativa/ZMenu/TextSequence?db=core;factorytype=Location;g=Os02g0510200;r=2:18236073-18238350;t=Os02t0510200-01;v=rs18704228;vf=4198779)G[G](http://plants.ensembl.org/Oryza_sativa/ZMenu/TextSequence?db=core;factorytype=Location;g=Os02g0510200;r=2:18236073-18238350;t=Os02t0510200-01;v=rs18704237;vf=4198780)CAA[C](http://plants.ensembl.org/Oryza_sativa/ZMenu/TextSequence?db=core;factorytype=Location;g=Os02g0510200;r=2:18236073-18238350;t=Os02t0510200-01;v=rs18704246;vf=4198781)[C](http://plants.ensembl.org/Oryza_sativa/ZMenu/TextSequence?db=core;factorytype=Location;g=Os02g0510200;r=2:18236073-18238350;t=Os02t0510200-01;v=rs18704255;vf=4198782)AACCT[C](http://plants.ensembl.org/Oryza_sativa/ZMenu/TextSequence?db=core;factorytype=Location;g=Os02g0510200;r=2:18236073-18238350;t=Os02t0510200-01;v=rs18704264;vf=4198783)GT[G](http://plants.ensembl.org/Oryza_sativa/ZMenu/TextSequence?db=core;factorytype=Location;g=Os02g0510200;r=2:18236073-18238350;t=Os02t0510200-01;v=rs18704273;vf=4198784)[T](http://plants.ensembl.org/Oryza_sativa/ZMenu/TextSequence?db=core;factorytype=Location;g=Os02g0510200;r=2:18236073-18238350;t=Os02t0510200-01;v=rs18704282;vf=4198785)[C](http://plants.ensembl.org/Oryza_sativa/ZMenu/TextSequence?db=core;factorytype=Location;g=Os02g0510200;r=2:18236073-18238350;t=Os02t0510200-01;v=rs18704291;vf=4198786)[C](http://plants.ensembl.org/Oryza_sativa/ZMenu/TextSequence?db=core;factorytype=Location;g=Os02g0510200;r=2:18236073-18238350;t=Os02t0510200-01;v=rs18704300;vf=4198787)G[C](http://plants.ensembl.org/Oryza_sativa/ZMenu/TextSequence?db=core;factorytype=Location;g=Os02g0510200;r=2:18236073-18238350;t=Os02t0510200-01;v=rs18704309;vf=4198788)[G](http://plants.ensembl.org/Oryza_sativa/ZMenu/TextSequence?db=core;factorytype=Location;g=Os02g0510200;r=2:18236073-18238350;t=Os02t0510200-01;v=rs18704318;vf=4198789)[C](http://plants.ensembl.org/Oryza_sativa/ZMenu/TextSequence?db=core;factorytype=Location;g=Os02g0510200;r=2:18236073-18238350;t=Os02t0510200-01;v=rs18704327;vf=4198790)T[C](http://plants.ensembl.org/Oryza_sativa/ZMenu/TextSequence?db=core;factorytype=Location;g=Os02g0510200;r=2:18236073-18238350;t=Os02t0510200-01;v=rs18704336;vf=4198791)G[C](http://plants.ensembl.org/Oryza_sativa/ZMenu/TextSequence?db=core;factorytype=Location;g=Os02g0510200;r=2:18236073-18238350;t=Os02t0510200-01;v=rs18704345;vf=4198792)[C](http://plants.ensembl.org/Oryza_sativa/ZMenu/TextSequence?db=core;factorytype=Location;g=Os02g0510200;r=2:18236073-18238350;t=Os02t0510200-01;v=rs18704354;vf=4198793)GA

A L L D S V P M V A I T G Q V **P** R R M I G T D **A** F Q E T P I V E V T R S I T

C[G](http://plants.ensembl.org/Oryza_sativa/ZMenu/TextSequence?db=core;factorytype=Location;g=Os02g0510200;r=2:18236073-18238350;t=Os02t0510200-01;v=rs18704363;vf=4198794)[C](http://plants.ensembl.org/Oryza_sativa/ZMenu/TextSequence?db=core;factorytype=Location;g=Os02g0510200;r=2:18236073-18238350;t=Os02t0510200-01;v=rs18704372;vf=4198795)[G](http://plants.ensembl.org/Oryza_sativa/ZMenu/TextSequence?db=core;factorytype=Location;g=Os02g0510200;r=2:18236073-18238350;t=Os02t0510200-01;v=rs18704381;vf=4198796)C[T](http://plants.ensembl.org/Oryza_sativa/ZMenu/TextSequence?db=core;factorytype=Location;g=Os02g0510200;r=2:18236073-18238350;t=Os02t0510200-01;v=rs18704390;vf=4198797)[G](http://plants.ensembl.org/Oryza_sativa/ZMenu/TextSequence?db=core;factorytype=Location;g=Os02g0510200;r=2:18236073-18238350;t=Os02t0510200-01;v=rs18704399;vf=4198798)C[T](http://plants.ensembl.org/Oryza_sativa/ZMenu/TextSequence?db=core;factorytype=Location;g=Os02g0510200;r=2:18236073-18238350;t=Os02t0510200-01;v=rs18704408;vf=4198799)CG[A](http://plants.ensembl.org/Oryza_sativa/ZMenu/TextSequence?db=core;factorytype=Location;g=Os02g0510200;r=2:18236073-18238350;t=Os02t0510200-01;v=rs18704417;vf=4198800)CT[C](http://plants.ensembl.org/Oryza_sativa/ZMenu/TextSequence?db=core;factorytype=Location;g=Os02g0510200;r=2:18236073-18238350;t=Os02t0510200-01;v=rs18704426;vf=4198801)CG[T](http://plants.ensembl.org/Oryza_sativa/ZMenu/TextSequence?db=core;factorytype=Location;g=Os02g0510200;r=2:18236073-18238350;t=Os02t0510200-01;v=rs18704435;vf=4198802)CC[C](http://plants.ensembl.org/Oryza_sativa/ZMenu/TextSequence?db=core;factorytype=Location;g=Os02g0510200;r=2:18236073-18238350;t=Os02t0510200-01;v=rs18704444;vf=4198803)[G](http://plants.ensembl.org/Oryza_sativa/ZMenu/TextSequence?db=core;factorytype=Location;g=Os02g0510200;r=2:18236073-18238350;t=Os02t0510200-01;v=rs18704453;vf=4198804)[A](http://plants.ensembl.org/Oryza_sativa/ZMenu/TextSequence?db=core;factorytype=Location;g=Os02g0510200;r=2:18236073-18238350;t=Os02t0510200-01;v=rs18704462;vf=4198805)T[G](http://plants.ensembl.org/Oryza_sativa/ZMenu/TextSequence?db=core;factorytype=Location;g=Os02g0510200;r=2:18236073-18238350;t=Os02t0510200-01;v=rs18704471;vf=4198806)[G](http://plants.ensembl.org/Oryza_sativa/ZMenu/TextSequence?db=core;factorytype=Location;g=Os02g0510200;r=2:18236073-18238350;t=Os02t0510200-01;v=rs18704480;vf=4198807)TCGC[C](http://plants.ensembl.org/Oryza_sativa/ZMenu/TextSequence?db=core;factorytype=Location;g=Os02g0510200;r=2:18236073-18238350;t=Os02t0510200-01;v=rs18704489;vf=4198808)[A](http://plants.ensembl.org/Oryza_sativa/ZMenu/TextSequence?db=core;factorytype=Location;g=Os02g0510200;r=2:18236073-18238350;t=Os02t0510200-01;v=rs18704498;vf=4198809)[T](http://plants.ensembl.org/Oryza_sativa/ZMenu/TextSequence?db=core;factorytype=Location;g=Os02g0510200;r=2:18236073-18238350;t=Os02t0510200-01;v=rs18704507;vf=4198810)C[A](http://plants.ensembl.org/Oryza_sativa/ZMenu/TextSequence?db=core;factorytype=Location;g=Os02g0510200;r=2:18236073-18238350;t=Os02t0510200-01;v=rs18704516;vf=4198811)CGGGCCAGGTC**CCC**CG[C](http://plants.ensembl.org/Oryza_sativa/ZMenu/TextSequence?db=core;factorytype=Location;g=Os02g0510200;r=2:18236073-18238350;t=Os02t0510200-01;v=rs18704525;vf=4198812)[C](http://plants.ensembl.org/Oryza_sativa/ZMenu/TextSequence?db=core;factorytype=Location;g=Os02g0510200;r=2:18236073-18238350;t=Os02t0510200-01;v=rs18704534;vf=4198813)GCATG[A](http://plants.ensembl.org/Oryza_sativa/ZMenu/TextSequence?db=core;factorytype=Location;g=Os02g0510200;r=2:18236073-18238350;t=Os02t0510200-01;v=rs18704543;vf=4198814)TCG[G](http://plants.ensembl.org/Oryza_sativa/ZMenu/TextSequence?db=core;factorytype=Location;g=Os02g0510200;r=2:18236073-18238350;t=Os02t0510200-01;v=rs18704552;vf=4198815)CACC[G](http://plants.ensembl.org/Oryza_sativa/ZMenu/TextSequence?db=core;factorytype=Location;g=Os02g0510200;r=2:18236073-18238350;t=Os02t0510200-01;v=rs18704561;vf=4198816)A[C](http://plants.ensembl.org/Oryza_sativa/ZMenu/TextSequence?db=core;factorytype=Location;g=Os02g0510200;r=2:18236073-18238350;t=Os02t0510200-01;v=rs18704570;vf=4198817)**G**[**C**](http://plants.ensembl.org/Oryza_sativa/ZMenu/TextSequence?db=core;factorytype=Location;g=Os02g0510200;r=2:18236073-18238350;t=Os02t0510200-01;v=rs18704579;vf=4198818)**C**[T](http://plants.ensembl.org/Oryza_sativa/ZMenu/TextSequence?db=core;factorytype=Location;g=Os02g0510200;r=2:18236073-18238350;t=Os02t0510200-01;v=rs18704588;vf=4198819)TCCAGGAGA[C](http://plants.ensembl.org/Oryza_sativa/ZMenu/TextSequence?db=core;factorytype=Location;g=Os02g0510200;r=2:18236073-18238350;t=Os02t0510200-01;v=rs18704597;vf=4198820)G[C](http://plants.ensembl.org/Oryza_sativa/ZMenu/TextSequence?db=core;factorytype=Location;g=Os02g0510200;r=2:18236073-18238350;t=Os02t0510200-01;v=rs18704606;vf=4198821)CC[A](http://plants.ensembl.org/Oryza_sativa/ZMenu/TextSequence?db=core;factorytype=Location;g=Os02g0510200;r=2:18236073-18238350;t=Os02t0510200-01;v=rs18704615;vf=4198822)T[A](http://plants.ensembl.org/Oryza_sativa/ZMenu/TextSequence?db=core;factorytype=Location;g=Os02g0510200;r=2:18236073-18238350;t=Os02t0510200-01;v=rs18704624;vf=4198823)GTC[G](http://plants.ensembl.org/Oryza_sativa/ZMenu/TextSequence?db=core;factorytype=Location;g=Os02g0510200;r=2:18236073-18238350;t=Os02t0510200-01;v=rs18704633;vf=4198824)[A](http://plants.ensembl.org/Oryza_sativa/ZMenu/TextSequence?db=core;factorytype=Location;g=Os02g0510200;r=2:18236073-18238350;t=Os02t0510200-01;v=rs18704642;vf=4198825)GG[T](http://plants.ensembl.org/Oryza_sativa/ZMenu/TextSequence?db=core;factorytype=Location;g=Os02g0510200;r=2:18236073-18238350;t=Os02t0510200-01;v=rs18704651;vf=4198826)CACCCG[C](http://plants.ensembl.org/Oryza_sativa/ZMenu/TextSequence?db=core;factorytype=Location;g=Os02g0510200;r=2:18236073-18238350;t=Os02t0510200-01;v=rs18704660;vf=4198827)T[C](http://plants.ensembl.org/Oryza_sativa/ZMenu/TextSequence?db=core;factorytype=Location;g=Os02g0510200;r=2:18236073-18238350;t=Os02t0510200-01;v=rs18704669;vf=4198828)[C](http://plants.ensembl.org/Oryza_sativa/ZMenu/TextSequence?db=core;factorytype=Location;g=Os02g0510200;r=2:18236073-18238350;t=Os02t0510200-01;v=rs18704678;vf=4198829)ATCACCA

K H N Y L V L D V E D I P R V I Q E A F F L A S S G R P G P V L V D I P K D I

[A](http://plants.ensembl.org/Oryza_sativa/ZMenu/TextSequence?db=core;factorytype=Location;g=Os02g0510200;r=2:18236073-18238350;t=Os02t0510200-01;v=rs18704687;vf=4198830)G[C](http://plants.ensembl.org/Oryza_sativa/ZMenu/TextSequence?db=core;factorytype=Location;g=Os02g0510200;r=2:18236073-18238350;t=Os02t0510200-01;v=rs18704696;vf=4198831)ACA[A](http://plants.ensembl.org/Oryza_sativa/ZMenu/TextSequence?db=core;factorytype=Location;g=Os02g0510200;r=2:18236073-18238350;t=Os02t0510200-01;v=rs18704705;vf=4198832)[T](http://plants.ensembl.org/Oryza_sativa/ZMenu/TextSequence?db=core;factorytype=Location;g=Os02g0510200;r=2:18236073-18238350;t=Os02t0510200-01;v=rs18704714;vf=4198833)[T](http://plants.ensembl.org/Oryza_sativa/ZMenu/TextSequence?db=core;factorytype=Location;g=Os02g0510200;r=2:18236073-18238350;t=Os02t0510200-01;v=rs18704723;vf=4198834)ACC[T](http://plants.ensembl.org/Oryza_sativa/ZMenu/TextSequence?db=core;factorytype=Location;g=Os02g0510200;r=2:18236073-18238350;t=Os02t0510200-01;v=rs18704732;vf=4198835)TG[T](http://plants.ensembl.org/Oryza_sativa/ZMenu/TextSequence?db=core;factorytype=Location;g=Os02g0510200;r=2:18236073-18238350;t=Os02t0510200-01;v=rs18704741;vf=4198836)CC[T](http://plants.ensembl.org/Oryza_sativa/ZMenu/TextSequence?db=core;factorytype=Location;g=Os02g0510200;r=2:18236073-18238350;t=Os02t0510200-01;v=rs18704750;vf=4198837)[T](http://plants.ensembl.org/Oryza_sativa/ZMenu/TextSequence?db=core;factorytype=Location;g=Os02g0510200;r=2:18236073-18238350;t=Os02t0510200-01;v=rs18704759;vf=4198838)G[A](http://plants.ensembl.org/Oryza_sativa/ZMenu/TextSequence?db=core;factorytype=Location;g=Os02g0510200;r=2:18236073-18238350;t=Os02t0510200-01;v=rs18704768;vf=4198839)[T](http://plants.ensembl.org/Oryza_sativa/ZMenu/TextSequence?db=core;factorytype=Location;g=Os02g0510200;r=2:18236073-18238350;t=Os02t0510200-01;v=rs18704777;vf=4198840)[G](http://plants.ensembl.org/Oryza_sativa/ZMenu/TextSequence?db=core;factorytype=Location;g=Os02g0510200;r=2:18236073-18238350;t=Os02t0510200-01;v=rs18704786;vf=4198841)[T](http://plants.ensembl.org/Oryza_sativa/ZMenu/TextSequence?db=core;factorytype=Location;g=Os02g0510200;r=2:18236073-18238350;t=Os02t0510200-01;v=rs18704795;vf=4198842)[G](http://plants.ensembl.org/Oryza_sativa/ZMenu/TextSequence?db=core;factorytype=Location;g=Os02g0510200;r=2:18236073-18238350;t=Os02t0510200-01;v=rs18704804;vf=4198843)[G](http://plants.ensembl.org/Oryza_sativa/ZMenu/TextSequence?db=core;factorytype=Location;g=Os02g0510200;r=2:18236073-18238350;t=Os02t0510200-01;v=rs18704813;vf=4198844)[A](http://plants.ensembl.org/Oryza_sativa/ZMenu/TextSequence?db=core;factorytype=Location;g=Os02g0510200;r=2:18236073-18238350;t=Os02t0510200-01;v=rs18704822;vf=4198845)[G](http://plants.ensembl.org/Oryza_sativa/ZMenu/TextSequence?db=core;factorytype=Location;g=Os02g0510200;r=2:18236073-18238350;t=Os02t0510200-01;v=rs18704831;vf=4198846)GACA[T](http://plants.ensembl.org/Oryza_sativa/ZMenu/TextSequence?db=core;factorytype=Location;g=Os02g0510200;r=2:18236073-18238350;t=Os02t0510200-01;v=rs18704840;vf=4198847)[C](http://plants.ensembl.org/Oryza_sativa/ZMenu/TextSequence?db=core;factorytype=Location;g=Os02g0510200;r=2:18236073-18238350;t=Os02t0510200-01;v=rs18704849;vf=4198848)[C](http://plants.ensembl.org/Oryza_sativa/ZMenu/TextSequence?db=core;factorytype=Location;g=Os02g0510200;r=2:18236073-18238350;t=Os02t0510200-01;v=rs18704858;vf=4198849)[C](http://plants.ensembl.org/Oryza_sativa/ZMenu/TextSequence?db=core;factorytype=Location;g=Os02g0510200;r=2:18236073-18238350;t=Os02t0510200-01;v=rs18704867;vf=4198850)[C](http://plants.ensembl.org/Oryza_sativa/ZMenu/TextSequence?db=core;factorytype=Location;g=Os02g0510200;r=2:18236073-18238350;t=Os02t0510200-01;v=rs18704876;vf=4198851)CGC[G](http://plants.ensembl.org/Oryza_sativa/ZMenu/TextSequence?db=core;factorytype=Location;g=Os02g0510200;r=2:18236073-18238350;t=Os02t0510200-01;v=rs18704885;vf=4198852)T[C](http://plants.ensembl.org/Oryza_sativa/ZMenu/TextSequence?db=core;factorytype=Location;g=Os02g0510200;r=2:18236073-18238350;t=Os02t0510200-01;v=rs18704894;vf=4198853)A[T](http://plants.ensembl.org/Oryza_sativa/ZMenu/TextSequence?db=core;factorytype=Location;g=Os02g0510200;r=2:18236073-18238350;t=Os02t0510200-01;v=rs18704903;vf=4198854)ACAGG[A](http://plants.ensembl.org/Oryza_sativa/ZMenu/TextSequence?db=core;factorytype=Location;g=Os02g0510200;r=2:18236073-18238350;t=Os02t0510200-01;v=rs18704912;vf=4198855)[A](http://plants.ensembl.org/Oryza_sativa/ZMenu/TextSequence?db=core;factorytype=Location;g=Os02g0510200;r=2:18236073-18238350;t=Os02t0510200-01;v=rs18704921;vf=4198856)[G](http://plants.ensembl.org/Oryza_sativa/ZMenu/TextSequence?db=core;factorytype=Location;g=Os02g0510200;r=2:18236073-18238350;t=Os02t0510200-01;v=rs18704930;vf=4198857)[C](http://plants.ensembl.org/Oryza_sativa/ZMenu/TextSequence?db=core;factorytype=Location;g=Os02g0510200;r=2:18236073-18238350;t=Os02t0510200-01;v=rs18704939;vf=4198858)C[T](http://plants.ensembl.org/Oryza_sativa/ZMenu/TextSequence?db=core;factorytype=Location;g=Os02g0510200;r=2:18236073-18238350;t=Os02t0510200-01;v=rs18704948;vf=4198859)TCT[T](http://plants.ensembl.org/Oryza_sativa/ZMenu/TextSequence?db=core;factorytype=Location;g=Os02g0510200;r=2:18236073-18238350;t=Os02t0510200-01;v=rs18704957;vf=4198860)[C](http://plants.ensembl.org/Oryza_sativa/ZMenu/TextSequence?db=core;factorytype=Location;g=Os02g0510200;r=2:18236073-18238350;t=Os02t0510200-01;v=rs18704966;vf=4198861)[C](http://plants.ensembl.org/Oryza_sativa/ZMenu/TextSequence?db=core;factorytype=Location;g=Os02g0510200;r=2:18236073-18238350;t=Os02t0510200-01;v=rs18704975;vf=4198862)T[C](http://plants.ensembl.org/Oryza_sativa/ZMenu/TextSequence?db=core;factorytype=Location;g=Os02g0510200;r=2:18236073-18238350;t=Os02t0510200-01;v=rs18704984;vf=4198863)GCG[T](http://plants.ensembl.org/Oryza_sativa/ZMenu/TextSequence?db=core;factorytype=Location;g=Os02g0510200;r=2:18236073-18238350;t=Os02t0510200-01;v=rs18704993;vf=4198864)[C](http://plants.ensembl.org/Oryza_sativa/ZMenu/TextSequence?db=core;factorytype=Location;g=Os02g0510200;r=2:18236073-18238350;t=Os02t0510200-01;v=rs18705002;vf=4198865)C[T](http://plants.ensembl.org/Oryza_sativa/ZMenu/TextSequence?db=core;factorytype=Location;g=Os02g0510200;r=2:18236073-18238350;t=Os02t0510200-01;v=rs18705011;vf=4198866)CGGGCCGTCCT[G](http://plants.ensembl.org/Oryza_sativa/ZMenu/TextSequence?db=core;factorytype=Location;g=Os02g0510200;r=2:18236073-18238350;t=Os02t0510200-01;v=rs18705020;vf=4198867)[G](http://plants.ensembl.org/Oryza_sativa/ZMenu/TextSequence?db=core;factorytype=Location;g=Os02g0510200;r=2:18236073-18238350;t=Os02t0510200-01;v=rs18705029;vf=4198868)[C](http://plants.ensembl.org/Oryza_sativa/ZMenu/TextSequence?db=core;factorytype=Location;g=Os02g0510200;r=2:18236073-18238350;t=Os02t0510200-01;v=rs18705038;vf=4198869)[C](http://plants.ensembl.org/Oryza_sativa/ZMenu/TextSequence?db=core;factorytype=Location;g=Os02g0510200;r=2:18236073-18238350;t=Os02t0510200-01;v=rs18705047;vf=4198870)CGGTG[C](http://plants.ensembl.org/Oryza_sativa/ZMenu/TextSequence?db=core;factorytype=Location;g=Os02g0510200;r=2:18236073-18238350;t=Os02t0510200-01;v=rs18705056;vf=4198871)TGG[T](http://plants.ensembl.org/Oryza_sativa/ZMenu/TextSequence?db=core;factorytype=Location;g=Os02g0510200;r=2:18236073-18238350;t=Os02t0510200-01;v=rs18705065;vf=4198872)[C](http://plants.ensembl.org/Oryza_sativa/ZMenu/TextSequence?db=core;factorytype=Location;g=Os02g0510200;r=2:18236073-18238350;t=Os02t0510200-01;v=rs18705074;vf=4198873)G[A](http://plants.ensembl.org/Oryza_sativa/ZMenu/TextSequence?db=core;factorytype=Location;g=Os02g0510200;r=2:18236073-18238350;t=Os02t0510200-01;v=rs18705083;vf=4198874)[C](http://plants.ensembl.org/Oryza_sativa/ZMenu/TextSequence?db=core;factorytype=Location;g=Os02g0510200;r=2:18236073-18238350;t=Os02t0510200-01;v=rs18705092;vf=4198875)[A](http://plants.ensembl.org/Oryza_sativa/ZMenu/TextSequence?db=core;factorytype=Location;g=Os02g0510200;r=2:18236073-18238350;t=Os02t0510200-01;v=rs18705101;vf=4198876)T[C](http://plants.ensembl.org/Oryza_sativa/ZMenu/TextSequence?db=core;factorytype=Location;g=Os02g0510200;r=2:18236073-18238350;t=Os02t0510200-01;v=rs18705110;vf=4198877)C[C](http://plants.ensembl.org/Oryza_sativa/ZMenu/TextSequence?db=core;factorytype=Location;g=Os02g0510200;r=2:18236073-18238350;t=Os02t0510200-01;v=rs18705119;vf=4198878)C[A](http://plants.ensembl.org/Oryza_sativa/ZMenu/TextSequence?db=core;factorytype=Location;g=Os02g0510200;r=2:18236073-18238350;t=Os02t0510200-01;v=rs18705128;vf=4198879)[A](http://plants.ensembl.org/Oryza_sativa/ZMenu/TextSequence?db=core;factorytype=Location;g=Os02g0510200;r=2:18236073-18238350;t=Os02t0510200-01;v=rs18705137;vf=4198880)GGAC[A](http://plants.ensembl.org/Oryza_sativa/ZMenu/TextSequence?db=core;factorytype=Location;g=Os02g0510200;r=2:18236073-18238350;t=Os02t0510200-01;v=rs18705146;vf=4198881)[T](http://plants.ensembl.org/Oryza_sativa/ZMenu/TextSequence?db=core;factorytype=Location;g=Os02g0510200;r=2:18236073-18238350;t=Os02t0510200-01;v=rs18705155;vf=4198882)[C](http://plants.ensembl.org/Oryza_sativa/ZMenu/TextSequence?db=core;factorytype=Location;g=Os02g0510200;r=2:18236073-18238350;t=Os02t0510200-01;v=rs18705164;vf=4198883)

Q Q Q M A V P V W D T S M N L P G Y I A R L P K P P A T E L L E Q V L R L V G

[C](http://plants.ensembl.org/Oryza_sativa/ZMenu/TextSequence?db=core;factorytype=Location;g=Os02g0510200;r=2:18236073-18238350;t=Os02t0510200-01;v=rs18705173;vf=4198884)[A](http://plants.ensembl.org/Oryza_sativa/ZMenu/TextSequence?db=core;factorytype=Location;g=Os02g0510200;r=2:18236073-18238350;t=Os02t0510200-01;v=rs18705182;vf=4198885)GCAGC[A](http://plants.ensembl.org/Oryza_sativa/ZMenu/TextSequence?db=core;factorytype=Location;g=Os02g0510200;r=2:18236073-18238350;t=Os02t0510200-01;v=rs18705191;vf=4198886)G[A](http://plants.ensembl.org/Oryza_sativa/ZMenu/TextSequence?db=core;factorytype=Location;g=Os02g0510200;r=2:18236073-18238350;t=Os02t0510200-01;v=rs18705200;vf=4198887)T[G](http://plants.ensembl.org/Oryza_sativa/ZMenu/TextSequence?db=core;factorytype=Location;g=Os02g0510200;r=2:18236073-18238350;t=Os02t0510200-01;v=rs18705209;vf=4198888)G[C](http://plants.ensembl.org/Oryza_sativa/ZMenu/TextSequence?db=core;factorytype=Location;g=Os02g0510200;r=2:18236073-18238350;t=Os02t0510200-01;v=rs18705218;vf=4198889)[C](http://plants.ensembl.org/Oryza_sativa/ZMenu/TextSequence?db=core;factorytype=Location;g=Os02g0510200;r=2:18236073-18238350;t=Os02t0510200-01;v=rs53977866;vf=4198890)G[T](http://plants.ensembl.org/Oryza_sativa/ZMenu/TextSequence?db=core;factorytype=Location;g=Os02g0510200;r=2:18236073-18238350;t=Os02t0510200-01;v=rs18705227;vf=4198891)GC[C](http://plants.ensembl.org/Oryza_sativa/ZMenu/TextSequence?db=core;factorytype=Location;g=Os02g0510200;r=2:18236073-18238350;t=Os02t0510200-01;v=rs18705236;vf=4198892)[G](http://plants.ensembl.org/Oryza_sativa/ZMenu/TextSequence?db=core;factorytype=Location;g=Os02g0510200;r=2:18236073-18238350;t=Os02t0510200-01;v=rs52853211;vf=4198893)G[T](http://plants.ensembl.org/Oryza_sativa/ZMenu/TextSequence?db=core;factorytype=Location;g=Os02g0510200;r=2:18236073-18238350;t=Os02t0510200-01;v=rs18705245;vf=4198894)CTGG[G](http://plants.ensembl.org/Oryza_sativa/ZMenu/TextSequence?db=core;factorytype=Location;g=Os02g0510200;r=2:18236073-18238350;t=Os02t0510200-01;v=rs18705254;vf=4198895)A[C](http://plants.ensembl.org/Oryza_sativa/ZMenu/TextSequence?db=core;factorytype=Location;g=Os02g0510200;r=2:18236073-18238350;t=Os02t0510200-01;v=rs18705263;vf=4198896)[A](http://plants.ensembl.org/Oryza_sativa/ZMenu/TextSequence?db=core;factorytype=Location;g=Os02g0510200;r=2:18236073-18238350;t=Os02t0510200-01;v=rs18705272;vf=4198897)CC[T](http://plants.ensembl.org/Oryza_sativa/ZMenu/TextSequence?db=core;factorytype=Location;g=Os02g0510200;r=2:18236073-18238350;t=Os02t0510200-01;v=rs18705281;vf=4198898)CG[A](http://plants.ensembl.org/Oryza_sativa/ZMenu/TextSequence?db=core;factorytype=Location;g=Os02g0510200;r=2:18236073-18238350;t=Os02t0510200-01;v=rs18705290;vf=4198899)TGAATC[T](http://plants.ensembl.org/Oryza_sativa/ZMenu/TextSequence?db=core;factorytype=Location;g=Os02g0510200;r=2:18236073-18238350;t=Os02t0510200-01;v=rs18705299;vf=4198900)ACC[A](http://plants.ensembl.org/Oryza_sativa/ZMenu/TextSequence?db=core;factorytype=Location;g=Os02g0510200;r=2:18236073-18238350;t=Os02t0510200-01;v=rs18705308;vf=4198901)[G](http://plants.ensembl.org/Oryza_sativa/ZMenu/TextSequence?db=core;factorytype=Location;g=Os02g0510200;r=2:18236073-18238350;t=Os02t0510200-01;v=rs18705317;vf=4198902)[G](http://plants.ensembl.org/Oryza_sativa/ZMenu/TextSequence?db=core;factorytype=Location;g=Os02g0510200;r=2:18236073-18238350;t=Os02t0510200-01;v=rs18705326;vf=4198903)[G](http://plants.ensembl.org/Oryza_sativa/ZMenu/TextSequence?db=core;factorytype=Location;g=Os02g0510200;r=2:18236073-18238350;t=Os02t0510200-01;v=rs18705335;vf=4198904)T[A](http://plants.ensembl.org/Oryza_sativa/ZMenu/TextSequence?db=core;factorytype=Location;g=Os02g0510200;r=2:18236073-18238350;t=Os02t0510200-01;v=rs18705344;vf=4198905)CAT[C](http://plants.ensembl.org/Oryza_sativa/ZMenu/TextSequence?db=core;factorytype=Location;g=Os02g0510200;r=2:18236073-18238350;t=Os02t0510200-01;v=rs18705353;vf=4198906)GCACGCCT[G](http://plants.ensembl.org/Oryza_sativa/ZMenu/TextSequence?db=core;factorytype=Location;g=Os02g0510200;r=2:18236073-18238350;t=Os02t0510200-01;v=rs18705362;vf=4198907)[C](http://plants.ensembl.org/Oryza_sativa/ZMenu/TextSequence?db=core;factorytype=Location;g=Os02g0510200;r=2:18236073-18238350;t=Os02t0510200-01;v=rs18705371;vf=4198908)[C](http://plants.ensembl.org/Oryza_sativa/ZMenu/TextSequence?db=core;factorytype=Location;g=Os02g0510200;r=2:18236073-18238350;t=Os02t0510200-01;v=rs18705380;vf=4198909)CAA[G](http://plants.ensembl.org/Oryza_sativa/ZMenu/TextSequence?db=core;factorytype=Location;g=Os02g0510200;r=2:18236073-18238350;t=Os02t0510200-01;v=rs18705389;vf=4198910)[C](http://plants.ensembl.org/Oryza_sativa/ZMenu/TextSequence?db=core;factorytype=Location;g=Os02g0510200;r=2:18236073-18238350;t=Os02t0510200-01;v=rs18705398;vf=4198911)[C](http://plants.ensembl.org/Oryza_sativa/ZMenu/TextSequence?db=core;factorytype=Location;g=Os02g0510200;r=2:18236073-18238350;t=Os02t0510200-01;v=rs18705407;vf=4198912)[A](http://plants.ensembl.org/Oryza_sativa/ZMenu/TextSequence?db=core;factorytype=Location;g=Os02g0510200;r=2:18236073-18238350;t=Os02t0510200-01;v=rs18705416;vf=4198913)CCCG[C](http://plants.ensembl.org/Oryza_sativa/ZMenu/TextSequence?db=core;factorytype=Location;g=Os02g0510200;r=2:18236073-18238350;t=Os02t0510200-01;v=rs18705425;vf=4198914)GACAGA[A](http://plants.ensembl.org/Oryza_sativa/ZMenu/TextSequence?db=core;factorytype=Location;g=Os02g0510200;r=2:18236073-18238350;t=Os02t0510200-01;v=rs18705434;vf=4198915)[T](http://plants.ensembl.org/Oryza_sativa/ZMenu/TextSequence?db=core;factorytype=Location;g=Os02g0510200;r=2:18236073-18238350;t=Os02t0510200-01;v=rs18705443;vf=4198916)[T](http://plants.ensembl.org/Oryza_sativa/ZMenu/TextSequence?db=core;factorytype=Location;g=Os02g0510200;r=2:18236073-18238350;t=Os02t0510200-01;v=rs18705452;vf=4198917)GCTTG[A](http://plants.ensembl.org/Oryza_sativa/ZMenu/TextSequence?db=core;factorytype=Location;g=Os02g0510200;r=2:18236073-18238350;t=Os02t0510200-01;v=rs18705461;vf=4198918)[G](http://plants.ensembl.org/Oryza_sativa/ZMenu/TextSequence?db=core;factorytype=Location;g=Os02g0510200;r=2:18236073-18238350;t=Os02t0510200-01;v=rs18705470;vf=4198919)C[A](http://plants.ensembl.org/Oryza_sativa/ZMenu/TextSequence?db=core;factorytype=Location;g=Os02g0510200;r=2:18236073-18238350;t=Os02t0510200-01;v=rs18705479;vf=4198920)[G](http://plants.ensembl.org/Oryza_sativa/ZMenu/TextSequence?db=core;factorytype=Location;g=Os02g0510200;r=2:18236073-18238350;t=Os02t0510200-01;v=rs18705488;vf=4198921)G[T](http://plants.ensembl.org/Oryza_sativa/ZMenu/TextSequence?db=core;factorytype=Location;g=Os02g0510200;r=2:18236073-18238350;t=Os02t0510200-01;v=rs18705497;vf=4198922)C[T](http://plants.ensembl.org/Oryza_sativa/ZMenu/TextSequence?db=core;factorytype=Location;g=Os02g0510200;r=2:18236073-18238350;t=Os02t0510200-01;v=rs18705506;vf=4198923)TGC[G](http://plants.ensembl.org/Oryza_sativa/ZMenu/TextSequence?db=core;factorytype=Location;g=Os02g0510200;r=2:18236073-18238350;t=Os02t0510200-01;v=rs18705515;vf=4198924)T[C](http://plants.ensembl.org/Oryza_sativa/ZMenu/TextSequence?db=core;factorytype=Location;g=Os02g0510200;r=2:18236073-18238350;t=Os02t0510200-01;v=rs18705524;vf=4198925)TGG[T](http://plants.ensembl.org/Oryza_sativa/ZMenu/TextSequence?db=core;factorytype=Location;g=Os02g0510200;r=2:18236073-18238350;t=Os02t0510200-01;v=rs18705533;vf=4198926)TGG

E S R R P I L Y V G G G C S A S G D E L R W F V E L T G I P V T T T L M G L

C[G](http://plants.ensembl.org/Oryza_sativa/ZMenu/TextSequence?db=core;factorytype=Location;g=Os02g0510200;r=2:18236073-18238350;t=Os02t0510200-01;v=rs18705542;vf=4198927)[A](http://plants.ensembl.org/Oryza_sativa/ZMenu/TextSequence?db=core;factorytype=Location;g=Os02g0510200;r=2:18236073-18238350;t=Os02t0510200-01;v=rs18705551;vf=4198928)G[T](http://plants.ensembl.org/Oryza_sativa/ZMenu/TextSequence?db=core;factorytype=Location;g=Os02g0510200;r=2:18236073-18238350;t=Os02t0510200-01;v=rs18705560;vf=4198929)CACGG[C](http://plants.ensembl.org/Oryza_sativa/ZMenu/TextSequence?db=core;factorytype=Location;g=Os02g0510200;r=2:18236073-18238350;t=Os02t0510200-01;v=rs18705569;vf=4198930)G[C](http://plants.ensembl.org/Oryza_sativa/ZMenu/TextSequence?db=core;factorytype=Location;g=Os02g0510200;r=2:18236073-18238350;t=Os02t0510200-01;v=rs18705578;vf=4198931)[C](http://plants.ensembl.org/Oryza_sativa/ZMenu/TextSequence?db=core;factorytype=Location;g=Os02g0510200;r=2:18236073-18238350;t=Os02t0510200-01;v=rs18705587;vf=4198932)CG[A](http://plants.ensembl.org/Oryza_sativa/ZMenu/TextSequence?db=core;factorytype=Location;g=Os02g0510200;r=2:18236073-18238350;t=Os02t0510200-01;v=rs18705596;vf=4198933)[T](http://plants.ensembl.org/Oryza_sativa/ZMenu/TextSequence?db=core;factorytype=Location;g=Os02g0510200;r=2:18236073-18238350;t=Os02t0510200-01;v=rs18705605;vf=4198934)[T](http://plants.ensembl.org/Oryza_sativa/ZMenu/TextSequence?db=core;factorytype=Location;g=Os02g0510200;r=2:18236073-18238350;t=Os02t0510200-01;v=rs18705614;vf=4198935)[C](http://plants.ensembl.org/Oryza_sativa/ZMenu/TextSequence?db=core;factorytype=Location;g=Os02g0510200;r=2:18236073-18238350;t=Os02t0510200-01;v=rs18705623;vf=4198936)TCTATG[T](http://plants.ensembl.org/Oryza_sativa/ZMenu/TextSequence?db=core;factorytype=Location;g=Os02g0510200;r=2:18236073-18238350;t=Os02t0510200-01;v=rs18705632;vf=4198937)CGGTGGTGGCTG[C](http://plants.ensembl.org/Oryza_sativa/ZMenu/TextSequence?db=core;factorytype=Location;g=Os02g0510200;r=2:18236073-18238350;t=Os02t0510200-01;v=rs18705641;vf=4198938)[T](http://plants.ensembl.org/Oryza_sativa/ZMenu/TextSequence?db=core;factorytype=Location;g=Os02g0510200;r=2:18236073-18238350;t=Os02t0510200-01;v=rs18705650;vf=4198939)CTG[C](http://plants.ensembl.org/Oryza_sativa/ZMenu/TextSequence?db=core;factorytype=Location;g=Os02g0510200;r=2:18236073-18238350;t=Os02t0510200-01;v=rs18705659;vf=4198940)[A](http://plants.ensembl.org/Oryza_sativa/ZMenu/TextSequence?db=core;factorytype=Location;g=Os02g0510200;r=2:18236073-18238350;t=Os02t0510200-01;v=rs18705668;vf=4198941)[T](http://plants.ensembl.org/Oryza_sativa/ZMenu/TextSequence?db=core;factorytype=Location;g=Os02g0510200;r=2:18236073-18238350;t=Os02t0510200-01;v=rs18705677;vf=4198942)CT[G](http://plants.ensembl.org/Oryza_sativa/ZMenu/TextSequence?db=core;factorytype=Location;g=Os02g0510200;r=2:18236073-18238350;t=Os02t0510200-01;v=rs18705686;vf=4198943)G[T](http://plants.ensembl.org/Oryza_sativa/ZMenu/TextSequence?db=core;factorytype=Location;g=Os02g0510200;r=2:18236073-18238350;t=Os02t0510200-01;v=rs18705695;vf=4198944)G[A](http://plants.ensembl.org/Oryza_sativa/ZMenu/TextSequence?db=core;factorytype=Location;g=Os02g0510200;r=2:18236073-18238350;t=Os02t0510200-01;v=rs18705704;vf=4198945)[C](http://plants.ensembl.org/Oryza_sativa/ZMenu/TextSequence?db=core;factorytype=Location;g=Os02g0510200;r=2:18236073-18238350;t=Os02t0510200-01;v=rs53056750;vf=4198946)[G](http://plants.ensembl.org/Oryza_sativa/ZMenu/TextSequence?db=core;factorytype=Location;g=Os02g0510200;r=2:18236073-18238350;t=Os02t0510200-01;v=rs18705713;vf=4198947)[A](http://plants.ensembl.org/Oryza_sativa/ZMenu/TextSequence?db=core;factorytype=Location;g=Os02g0510200;r=2:18236073-18238350;t=Os02t0510200-01;v=rs18705722;vf=4198948)[A](http://plants.ensembl.org/Oryza_sativa/ZMenu/TextSequence?db=core;factorytype=Location;g=Os02g0510200;r=2:18236073-18238350;t=Os02t0510200-01;v=rs18705731;vf=4198949)TTG[C](http://plants.ensembl.org/Oryza_sativa/ZMenu/TextSequence?db=core;factorytype=Location;g=Os02g0510200;r=2:18236073-18238350;t=Os02t0510200-01;v=rs18705740;vf=4198950)GC[T](http://plants.ensembl.org/Oryza_sativa/ZMenu/TextSequence?db=core;factorytype=Location;g=Os02g0510200;r=2:18236073-18238350;t=Os02t0510200-01;v=rs53818773;vf=4198951)GGTTTG[T](http://plants.ensembl.org/Oryza_sativa/ZMenu/TextSequence?db=core;factorytype=Location;g=Os02g0510200;r=2:18236073-18238350;t=Os02t0510200-01;v=rs18705749;vf=4198952)TGA[G](http://plants.ensembl.org/Oryza_sativa/ZMenu/TextSequence?db=core;factorytype=Location;g=Os02g0510200;r=2:18236073-18238350;t=Os02t0510200-01;v=rs18705758;vf=4198953)CTGAC[T](http://plants.ensembl.org/Oryza_sativa/ZMenu/TextSequence?db=core;factorytype=Location;g=Os02g0510200;r=2:18236073-18238350;t=Os02t0510200-01;v=rs18705767;vf=4198954)GG[T](http://plants.ensembl.org/Oryza_sativa/ZMenu/TextSequence?db=core;factorytype=Location;g=Os02g0510200;r=2:18236073-18238350;t=Os02t0510200-01;v=rs18705776;vf=4198955)A[T](http://plants.ensembl.org/Oryza_sativa/ZMenu/TextSequence?db=core;factorytype=Location;g=Os02g0510200;r=2:18236073-18238350;t=Os02t0510200-01;v=rs18705785;vf=4198956)[C](http://plants.ensembl.org/Oryza_sativa/ZMenu/TextSequence?db=core;factorytype=Location;g=Os02g0510200;r=2:18236073-18238350;t=Os02t0510200-01;v=rs18705794;vf=4198957)[C](http://plants.ensembl.org/Oryza_sativa/ZMenu/TextSequence?db=core;factorytype=Location;g=Os02g0510200;r=2:18236073-18238350;t=Os02t0510200-01;v=rs18705803;vf=4198958)[C](http://plants.ensembl.org/Oryza_sativa/ZMenu/TextSequence?db=core;factorytype=Location;g=Os02g0510200;r=2:18236073-18238350;t=Os02t0510200-01;v=rs18705812;vf=4198959)[A](http://plants.ensembl.org/Oryza_sativa/ZMenu/TextSequence?db=core;factorytype=Location;g=Os02g0510200;r=2:18236073-18238350;t=Os02t0510200-01;v=rs18705821;vf=4198960)G[T](http://plants.ensembl.org/Oryza_sativa/ZMenu/TextSequence?db=core;factorytype=Location;g=Os02g0510200;r=2:18236073-18238350;t=Os02t0510200-01;v=rs18705830;vf=4198961)[T](http://plants.ensembl.org/Oryza_sativa/ZMenu/TextSequence?db=core;factorytype=Location;g=Os02g0510200;r=2:18236073-18238350;t=Os02t0510200-01;v=rs18705839;vf=4198962)[A](http://plants.ensembl.org/Oryza_sativa/ZMenu/TextSequence?db=core;factorytype=Location;g=Os02g0510200;r=2:18236073-18238350;t=Os02t0510200-01;v=rs18705848;vf=4198963)C[A](http://plants.ensembl.org/Oryza_sativa/ZMenu/TextSequence?db=core;factorytype=Location;g=Os02g0510200;r=2:18236073-18238350;t=Os02t0510200-01;v=rs18705857;vf=4198964)[A](http://plants.ensembl.org/Oryza_sativa/ZMenu/TextSequence?db=core;factorytype=Location;g=Os02g0510200;r=2:18236073-18238350;t=Os02t0510200-01;v=rs18705866;vf=4198965)[C](http://plants.ensembl.org/Oryza_sativa/ZMenu/TextSequence?db=core;factorytype=Location;g=Os02g0510200;r=2:18236073-18238350;t=Os02t0510200-01;v=rs18705875;vf=4198966)[C](http://plants.ensembl.org/Oryza_sativa/ZMenu/TextSequence?db=core;factorytype=Location;g=Os02g0510200;r=2:18236073-18238350;t=Os02t0510200-01;v=rs18705884;vf=4198967)[A](http://plants.ensembl.org/Oryza_sativa/ZMenu/TextSequence?db=core;factorytype=Location;g=Os02g0510200;r=2:18236073-18238350;t=Os02t0510200-01;v=rs18705893;vf=4198968)C[T](http://plants.ensembl.org/Oryza_sativa/ZMenu/TextSequence?db=core;factorytype=Location;g=Os02g0510200;r=2:18236073-18238350;t=Os02t0510200-01;v=rs18705902;vf=4198969)[C](http://plants.ensembl.org/Oryza_sativa/ZMenu/TextSequence?db=core;factorytype=Location;g=Os02g0510200;r=2:18236073-18238350;t=Os02t0510200-01;v=rs18705911;vf=4198970)[T](http://plants.ensembl.org/Oryza_sativa/ZMenu/TextSequence?db=core;factorytype=Location;g=Os02g0510200;r=2:18236073-18238350;t=Os02t0510200-01;v=rs18705920;vf=4198971)[G](http://plants.ensembl.org/Oryza_sativa/ZMenu/TextSequence?db=core;factorytype=Location;g=Os02g0510200;r=2:18236073-18238350;t=Os02t0510200-01;v=rs18705929;vf=4198972)ATGGGCC[T](http://plants.ensembl.org/Oryza_sativa/ZMenu/TextSequence?db=core;factorytype=Location;g=Os02g0510200;r=2:18236073-18238350;t=Os02t0510200-01;v=rs18705938;vf=4198973)[C](http://plants.ensembl.org/Oryza_sativa/ZMenu/TextSequence?db=core;factorytype=Location;g=Os02g0510200;r=2:18236073-18238350;t=Os02t0510200-01;v=rs18705947;vf=4198974)G

G N F P S D D P L S L R M L G M H G T V Y A N Y A V D K A D L L L A F G V R F

GCA[A](http://plants.ensembl.org/Oryza_sativa/ZMenu/TextSequence?db=core;factorytype=Location;g=Os02g0510200;r=2:18236073-18238350;t=Os02t0510200-01;v=rs18705956;vf=4198975)T[T](http://plants.ensembl.org/Oryza_sativa/ZMenu/TextSequence?db=core;factorytype=Location;g=Os02g0510200;r=2:18236073-18238350;t=Os02t0510200-01;v=rs18705965;vf=4198976)T[C](http://plants.ensembl.org/Oryza_sativa/ZMenu/TextSequence?db=core;factorytype=Location;g=Os02g0510200;r=2:18236073-18238350;t=Os02t0510200-01;v=rs18705974;vf=4198977)C[C](http://plants.ensembl.org/Oryza_sativa/ZMenu/TextSequence?db=core;factorytype=Location;g=Os02g0510200;r=2:18236073-18238350;t=Os02t0510200-01;v=rs18705983;vf=4198978)CAGTGA[C](http://plants.ensembl.org/Oryza_sativa/ZMenu/TextSequence?db=core;factorytype=Location;g=Os02g0510200;r=2:18236073-18238350;t=Os02t0510200-01;v=rs54033222;vf=4198979)GA[C](http://plants.ensembl.org/Oryza_sativa/ZMenu/TextSequence?db=core;factorytype=Location;g=Os02g0510200;r=2:18236073-18238350;t=Os02t0510200-01;v=rs53212221;vf=4198980)CC[G](http://plants.ensembl.org/Oryza_sativa/ZMenu/TextSequence?db=core;factorytype=Location;g=Os02g0510200;r=2:18236073-18238350;t=Os02t0510200-01;v=rs18705992;vf=4198981)TTG[T](http://plants.ensembl.org/Oryza_sativa/ZMenu/TextSequence?db=core;factorytype=Location;g=Os02g0510200;r=2:18236073-18238350;t=Os02t0510200-01;v=rs18706001;vf=4198982)[C](http://plants.ensembl.org/Oryza_sativa/ZMenu/TextSequence?db=core;factorytype=Location;g=Os02g0510200;r=2:18236073-18238350;t=Os02t0510200-01;v=rs18706010;vf=4198983)CC[T](http://plants.ensembl.org/Oryza_sativa/ZMenu/TextSequence?db=core;factorytype=Location;g=Os02g0510200;r=2:18236073-18238350;t=Os02t0510200-01;v=rs18706019;vf=4198984)GC[G](http://plants.ensembl.org/Oryza_sativa/ZMenu/TextSequence?db=core;factorytype=Location;g=Os02g0510200;r=2:18236073-18238350;t=Os02t0510200-01;v=rs18706028;vf=4198985)[C](http://plants.ensembl.org/Oryza_sativa/ZMenu/TextSequence?db=core;factorytype=Location;g=Os02g0510200;r=2:18236073-18238350;t=Os02t0510200-01;v=rs18706037;vf=4198986)ATG[C](http://plants.ensembl.org/Oryza_sativa/ZMenu/TextSequence?db=core;factorytype=Location;g=Os02g0510200;r=2:18236073-18238350;t=Os02t0510200-01;v=rs18706046;vf=4198987)TTGG[G](http://plants.ensembl.org/Oryza_sativa/ZMenu/TextSequence?db=core;factorytype=Location;g=Os02g0510200;r=2:18236073-18238350;t=Os02t0510200-01;v=rs18706055;vf=4198988)A[T](http://plants.ensembl.org/Oryza_sativa/ZMenu/TextSequence?db=core;factorytype=Location;g=Os02g0510200;r=2:18236073-18238350;t=Os02t0510200-01;v=rs18706064;vf=4198989)GCA[T](http://plants.ensembl.org/Oryza_sativa/ZMenu/TextSequence?db=core;factorytype=Location;g=Os02g0510200;r=2:18236073-18238350;t=Os02t0510200-01;v=rs18706073;vf=4198990)G[G](http://plants.ensembl.org/Oryza_sativa/ZMenu/TextSequence?db=core;factorytype=Location;g=Os02g0510200;r=2:18236073-18238350;t=Os02t0510200-01;v=rs18706082;vf=4198991)[C](http://plants.ensembl.org/Oryza_sativa/ZMenu/TextSequence?db=core;factorytype=Location;g=Os02g0510200;r=2:18236073-18238350;t=Os02t0510200-01;v=rs18706091;vf=4198992)AC[G](http://plants.ensembl.org/Oryza_sativa/ZMenu/TextSequence?db=core;factorytype=Location;g=Os02g0510200;r=2:18236073-18238350;t=Os02t0510200-01;v=rs18706100;vf=4198993)[G](http://plants.ensembl.org/Oryza_sativa/ZMenu/TextSequence?db=core;factorytype=Location;g=Os02g0510200;r=2:18236073-18238350;t=Os02t0510200-01;v=rs18706109;vf=4198994)[T](http://plants.ensembl.org/Oryza_sativa/ZMenu/TextSequence?db=core;factorytype=Location;g=Os02g0510200;r=2:18236073-18238350;t=Os02t0510200-01;v=rs18706118;vf=4198995)[G](http://plants.ensembl.org/Oryza_sativa/ZMenu/TextSequence?db=core;factorytype=Location;g=Os02g0510200;r=2:18236073-18238350;t=Os02t0510200-01;v=rs18706127;vf=4198996)T[A](http://plants.ensembl.org/Oryza_sativa/ZMenu/TextSequence?db=core;factorytype=Location;g=Os02g0510200;r=2:18236073-18238350;t=Os02t0510200-01;v=rs18706136;vf=4198997)[C](http://plants.ensembl.org/Oryza_sativa/ZMenu/TextSequence?db=core;factorytype=Location;g=Os02g0510200;r=2:18236073-18238350;t=Os02t0510200-01;v=rs18706145;vf=4198998)GCAA[A](http://plants.ensembl.org/Oryza_sativa/ZMenu/TextSequence?db=core;factorytype=Location;g=Os02g0510200;r=2:18236073-18238350;t=Os02t0510200-01;v=rs18706154;vf=4198999)TT[A](http://plants.ensembl.org/Oryza_sativa/ZMenu/TextSequence?db=core;factorytype=Location;g=Os02g0510200;r=2:18236073-18238350;t=Os02t0510200-01;v=rs18706163;vf=4199000)[T](http://plants.ensembl.org/Oryza_sativa/ZMenu/TextSequence?db=core;factorytype=Location;g=Os02g0510200;r=2:18236073-18238350;t=Os02t0510200-01;v=rs18706172;vf=4199001)GC[C](http://plants.ensembl.org/Oryza_sativa/ZMenu/TextSequence?db=core;factorytype=Location;g=Os02g0510200;r=2:18236073-18238350;t=Os02t0510200-01;v=rs18706181;vf=4199002)G[T](http://plants.ensembl.org/Oryza_sativa/ZMenu/TextSequence?db=core;factorytype=Location;g=Os02g0510200;r=2:18236073-18238350;t=Os02t0510200-01;v=rs18706190;vf=4199003)[G](http://plants.ensembl.org/Oryza_sativa/ZMenu/TextSequence?db=core;factorytype=Location;g=Os02g0510200;r=2:18236073-18238350;t=Os02t0510200-01;v=rs18706199;vf=4199004)G[A](http://plants.ensembl.org/Oryza_sativa/ZMenu/TextSequence?db=core;factorytype=Location;g=Os02g0510200;r=2:18236073-18238350;t=Os02t0510200-01;v=rs18706208;vf=4199005)TAAG[G](http://plants.ensembl.org/Oryza_sativa/ZMenu/TextSequence?db=core;factorytype=Location;g=Os02g0510200;r=2:18236073-18238350;t=Os02t0510200-01;v=rs18706217;vf=4199006)[C](http://plants.ensembl.org/Oryza_sativa/ZMenu/TextSequence?db=core;factorytype=Location;g=Os02g0510200;r=2:18236073-18238350;t=Os02t0510200-01;v=rs18706226;vf=4199007)TGACCTGTT[G](http://plants.ensembl.org/Oryza_sativa/ZMenu/TextSequence?db=core;factorytype=Location;g=Os02g0510200;r=2:18236073-18238350;t=Os02t0510200-01;v=rs18706235;vf=4199008)C[T](http://plants.ensembl.org/Oryza_sativa/ZMenu/TextSequence?db=core;factorytype=Location;g=Os02g0510200;r=2:18236073-18238350;t=Os02t0510200-01;v=rs18706244;vf=4199009)[T](http://plants.ensembl.org/Oryza_sativa/ZMenu/TextSequence?db=core;factorytype=Location;g=Os02g0510200;r=2:18236073-18238350;t=Os02t0510200-01;v=rs18706252;vf=4199010)G[C](http://plants.ensembl.org/Oryza_sativa/ZMenu/TextSequence?db=core;factorytype=Location;g=Os02g0510200;r=2:18236073-18238350;t=Os02t0510200-01;v=rs18706261;vf=4199011)[G](http://plants.ensembl.org/Oryza_sativa/ZMenu/TextSequence?db=core;factorytype=Location;g=Os02g0510200;r=2:18236073-18238350;t=Os02t0510200-01;v=rs18706270;vf=4199012)TT[T](http://plants.ensembl.org/Oryza_sativa/ZMenu/TextSequence?db=core;factorytype=Location;g=Os02g0510200;r=2:18236073-18238350;t=Os02t0510200-01;v=rs18706279;vf=4199013)[G](http://plants.ensembl.org/Oryza_sativa/ZMenu/TextSequence?db=core;factorytype=Location;g=Os02g0510200;r=2:18236073-18238350;t=Os02t0510200-01;v=rs18706288;vf=4199014)[G](http://plants.ensembl.org/Oryza_sativa/ZMenu/TextSequence?db=core;factorytype=Location;g=Os02g0510200;r=2:18236073-18238350;t=Os02t0510200-01;v=rs18706297;vf=4199015)[T](http://plants.ensembl.org/Oryza_sativa/ZMenu/TextSequence?db=core;factorytype=Location;g=Os02g0510200;r=2:18236073-18238350;t=Os02t0510200-01;v=rs18706306;vf=4199016)G[T](http://plants.ensembl.org/Oryza_sativa/ZMenu/TextSequence?db=core;factorytype=Location;g=Os02g0510200;r=2:18236073-18238350;t=Os02t0510200-01;v=rs18706315;vf=4199017)[G](http://plants.ensembl.org/Oryza_sativa/ZMenu/TextSequence?db=core;factorytype=Location;g=Os02g0510200;r=2:18236073-18238350;t=Os02t0510200-01;v=rs18706324;vf=4199018)C[G](http://plants.ensembl.org/Oryza_sativa/ZMenu/TextSequence?db=core;factorytype=Location;g=Os02g0510200;r=2:18236073-18238350;t=Os02t0510200-01;v=rs18706333;vf=4199019)G[T](http://plants.ensembl.org/Oryza_sativa/ZMenu/TextSequence?db=core;factorytype=Location;g=Os02g0510200;r=2:18236073-18238350;t=Os02t0510200-01;v=rs18706342;vf=4199020)T[T](http://plants.ensembl.org/Oryza_sativa/ZMenu/TextSequence?db=core;factorytype=Location;g=Os02g0510200;r=2:18236073-18238350;t=Os02t0510200-01;v=rs18706351;vf=4199021)

D **D** **R** V T G K I E A F A S R A K I V H I D I D P A E I G K N K Q P H V S I C A

[G](http://plants.ensembl.org/Oryza_sativa/ZMenu/TextSequence?db=core;factorytype=Location;g=Os02g0510200;r=2:18236073-18238350;t=Os02t0510200-01;v=rs18706360;vf=4199022)A[T](http://plants.ensembl.org/Oryza_sativa/ZMenu/TextSequence?db=core;factorytype=Location;g=Os02g0510200;r=2:18236073-18238350;t=Os02t0510200-01;v=rs18706369;vf=4199023)[**G**](http://plants.ensembl.org/Oryza_sativa/ZMenu/TextSequence?db=core;factorytype=Location;g=Os02g0510200;r=2:18236073-18238350;t=Os02t0510200-01;v=rs18706378;vf=4199024)**A**[**T**](http://plants.ensembl.org/Oryza_sativa/ZMenu/TextSequence?db=core;factorytype=Location;g=Os02g0510200;r=2:18236073-18238350;t=Os02t0510200-01;v=rs18706387;vf=4199025)[**C**](http://plants.ensembl.org/Oryza_sativa/ZMenu/TextSequence?db=core;factorytype=Location;g=Os02g0510200;r=2:18236073-18238350;t=Os02t0510200-01;v=rs18706396;vf=4199026)**G**[**T**](http://plants.ensembl.org/Oryza_sativa/ZMenu/TextSequence?db=core;factorytype=Location;g=Os02g0510200;r=2:18236073-18238350;t=Os02t0510200-01;v=rs18706405;vf=4199027)[G](http://plants.ensembl.org/Oryza_sativa/ZMenu/TextSequence?db=core;factorytype=Location;g=Os02g0510200;r=2:18236073-18238350;t=Os02t0510200-01;v=rs18706414;vf=4199028)[T](http://plants.ensembl.org/Oryza_sativa/ZMenu/TextSequence?db=core;factorytype=Location;g=Os02g0510200;r=2:18236073-18238350;t=Os02t0510200-01;v=rs18706423;vf=4199029)[G](http://plants.ensembl.org/Oryza_sativa/ZMenu/TextSequence?db=core;factorytype=Location;g=Os02g0510200;r=2:18236073-18238350;t=Os02t0510200-01;v=rs18706432;vf=4199030)AC[A](http://plants.ensembl.org/Oryza_sativa/ZMenu/TextSequence?db=core;factorytype=Location;g=Os02g0510200;r=2:18236073-18238350;t=Os02t0510200-01;v=rs53701665;vf=4199031)[G](http://plants.ensembl.org/Oryza_sativa/ZMenu/TextSequence?db=core;factorytype=Location;g=Os02g0510200;r=2:18236073-18238350;t=Os02t0510200-01;v=rs18706441;vf=4199032)[G](http://plants.ensembl.org/Oryza_sativa/ZMenu/TextSequence?db=core;factorytype=Location;g=Os02g0510200;r=2:18236073-18238350;t=Os02t0510200-01;v=rs18706450;vf=4199033)GA[A](http://plants.ensembl.org/Oryza_sativa/ZMenu/TextSequence?db=core;factorytype=Location;g=Os02g0510200;r=2:18236073-18238350;t=Os02t0510200-01;v=rs18706459;vf=4199034)AA[T](http://plants.ensembl.org/Oryza_sativa/ZMenu/TextSequence?db=core;factorytype=Location;g=Os02g0510200;r=2:18236073-18238350;t=Os02t0510200-01;v=rs18706468;vf=4199035)T[G](http://plants.ensembl.org/Oryza_sativa/ZMenu/TextSequence?db=core;factorytype=Location;g=Os02g0510200;r=2:18236073-18238350;t=Os02t0510200-01;v=rs18706477;vf=4199036)[A](http://plants.ensembl.org/Oryza_sativa/ZMenu/TextSequence?db=core;factorytype=Location;g=Os02g0510200;r=2:18236073-18238350;t=Os02t0510200-01;v=rs18706486;vf=4199037)[G](http://plants.ensembl.org/Oryza_sativa/ZMenu/TextSequence?db=core;factorytype=Location;g=Os02g0510200;r=2:18236073-18238350;t=Os02t0510200-01;v=rs18706495;vf=4199038)G[C](http://plants.ensembl.org/Oryza_sativa/ZMenu/TextSequence?db=core;factorytype=Location;g=Os02g0510200;r=2:18236073-18238350;t=Os02t0510200-01;v=rs18706504;vf=4199039)TTTT[G](http://plants.ensembl.org/Oryza_sativa/ZMenu/TextSequence?db=core;factorytype=Location;g=Os02g0510200;r=2:18236073-18238350;t=Os02t0510200-01;v=rs18706513;vf=4199040)[C](http://plants.ensembl.org/Oryza_sativa/ZMenu/TextSequence?db=core;factorytype=Location;g=Os02g0510200;r=2:18236073-18238350;t=Os02t0510200-01;v=rs18706522;vf=4199041)[A](http://plants.ensembl.org/Oryza_sativa/ZMenu/TextSequence?db=core;factorytype=Location;g=Os02g0510200;r=2:18236073-18238350;t=Os02t0510200-01;v=rs18706531;vf=4199042)AGCA[G](http://plants.ensembl.org/Oryza_sativa/ZMenu/TextSequence?db=core;factorytype=Location;g=Os02g0510200;r=2:18236073-18238350;t=Os02t0510200-01;v=rs18706540;vf=4199043)GGCCAAGA[T](http://plants.ensembl.org/Oryza_sativa/ZMenu/TextSequence?db=core;factorytype=Location;g=Os02g0510200;r=2:18236073-18238350;t=Os02t0510200-01;v=rs18706549;vf=4199044)TGTG[C](http://plants.ensembl.org/Oryza_sativa/ZMenu/TextSequence?db=core;factorytype=Location;g=Os02g0510200;r=2:18236073-18238350;t=Os02t0510200-01;v=rs18706558;vf=4199045)AC[A](http://plants.ensembl.org/Oryza_sativa/ZMenu/TextSequence?db=core;factorytype=Location;g=Os02g0510200;r=2:18236073-18238350;t=Os02t0510200-01;v=rs18706567;vf=4199046)[T](http://plants.ensembl.org/Oryza_sativa/ZMenu/TextSequence?db=core;factorytype=Location;g=Os02g0510200;r=2:18236073-18238350;t=Os02t0510200-01;v=rs18706576;vf=4199047)TGACATTGA[T](http://plants.ensembl.org/Oryza_sativa/ZMenu/TextSequence?db=core;factorytype=Location;g=Os02g0510200;r=2:18236073-18238350;t=Os02t0510200-01;v=rs18706585;vf=4199048)[C](http://plants.ensembl.org/Oryza_sativa/ZMenu/TextSequence?db=core;factorytype=Location;g=Os02g0510200;r=2:18236073-18238350;t=Os02t0510200-01;v=rs18706594;vf=4199049)CAGC[A](http://plants.ensembl.org/Oryza_sativa/ZMenu/TextSequence?db=core;factorytype=Location;g=Os02g0510200;r=2:18236073-18238350;t=Os02t0510200-01;v=rs53223042;vf=4199050)GAG[A](http://plants.ensembl.org/Oryza_sativa/ZMenu/TextSequence?db=core;factorytype=Location;g=Os02g0510200;r=2:18236073-18238350;t=Os02t0510200-01;v=rs18706603;vf=4199051)TT[G](http://plants.ensembl.org/Oryza_sativa/ZMenu/TextSequence?db=core;factorytype=Location;g=Os02g0510200;r=2:18236073-18238350;t=Os02t0510200-01;v=rs18706612;vf=4199052)[G](http://plants.ensembl.org/Oryza_sativa/ZMenu/TextSequence?db=core;factorytype=Location;g=Os02g0510200;r=2:18236073-18238350;t=Os02t0510200-01;v=rs18706621;vf=4199053)[A](http://plants.ensembl.org/Oryza_sativa/ZMenu/TextSequence?db=core;factorytype=Location;g=Os02g0510200;r=2:18236073-18238350;t=Os02t0510200-01;v=rs18706630;vf=4199054)[A](http://plants.ensembl.org/Oryza_sativa/ZMenu/TextSequence?db=core;factorytype=Location;g=Os02g0510200;r=2:18236073-18238350;t=Os02t0510200-01;v=rs18706639;vf=4199055)[A](http://plants.ensembl.org/Oryza_sativa/ZMenu/TextSequence?db=core;factorytype=Location;g=Os02g0510200;r=2:18236073-18238350;t=Os02t0510200-01;v=rs18706648;vf=4199056)G[A](http://plants.ensembl.org/Oryza_sativa/ZMenu/TextSequence?db=core;factorytype=Location;g=Os02g0510200;r=2:18236073-18238350;t=Os02t0510200-01;v=rs18706657;vf=4199057)[A](http://plants.ensembl.org/Oryza_sativa/ZMenu/TextSequence?db=core;factorytype=Location;g=Os02g0510200;r=2:18236073-18238350;t=Os02t0510200-01;v=rs18706666;vf=4199058)[C](http://plants.ensembl.org/Oryza_sativa/ZMenu/TextSequence?db=core;factorytype=Location;g=Os02g0510200;r=2:18236073-18238350;t=Os02t0510200-01;v=rs18706675;vf=4199059)[A](http://plants.ensembl.org/Oryza_sativa/ZMenu/TextSequence?db=core;factorytype=Location;g=Os02g0510200;r=2:18236073-18238350;t=Os02t0510200-01;v=rs18706684;vf=4199060)AGCAA[C](http://plants.ensembl.org/Oryza_sativa/ZMenu/TextSequence?db=core;factorytype=Location;g=Os02g0510200;r=2:18236073-18238350;t=Os02t0510200-01;v=rs18706693;vf=4199061)CAC[A](http://plants.ensembl.org/Oryza_sativa/ZMenu/TextSequence?db=core;factorytype=Location;g=Os02g0510200;r=2:18236073-18238350;t=Os02t0510200-01;v=rs18706702;vf=4199062)T[G](http://plants.ensembl.org/Oryza_sativa/ZMenu/TextSequence?db=core;factorytype=Location;g=Os02g0510200;r=2:18236073-18238350;t=Os02t0510200-01;v=rs18706711;vf=4199063)TGT[C](http://plants.ensembl.org/Oryza_sativa/ZMenu/TextSequence?db=core;factorytype=Location;g=Os02g0510200;r=2:18236073-18238350;t=Os02t0510200-01;v=rs18706720;vf=4199064)AA[T](http://plants.ensembl.org/Oryza_sativa/ZMenu/TextSequence?db=core;factorytype=Location;g=Os02g0510200;r=2:18236073-18238350;t=Os02t0510200-01;v=rs18706729;vf=4199065)[T](http://plants.ensembl.org/Oryza_sativa/ZMenu/TextSequence?db=core;factorytype=Location;g=Os02g0510200;r=2:18236073-18238350;t=Os02t0510200-01;v=rs18706738;vf=4199066)TG[C](http://plants.ensembl.org/Oryza_sativa/ZMenu/TextSequence?db=core;factorytype=Location;g=Os02g0510200;r=2:18236073-18238350;t=Os02t0510200-01;v=rs18706747;vf=4199067)[G](http://plants.ensembl.org/Oryza_sativa/ZMenu/TextSequence?db=core;factorytype=Location;g=Os02g0510200;r=2:18236073-18238350;t=Os02t0510200-01;v=rs18706756;vf=4199068)C

D V K L A L Q G L N A L L Q Q S T T K T S S D F S A W H N E L D Q Q K R E F

[A](http://plants.ensembl.org/Oryza_sativa/ZMenu/TextSequence?db=core;factorytype=Location;g=Os02g0510200;r=2:18236073-18238350;t=Os02t0510200-01;v=rs18706765;vf=4199069)[G](http://plants.ensembl.org/Oryza_sativa/ZMenu/TextSequence?db=core;factorytype=Location;g=Os02g0510200;r=2:18236073-18238350;t=Os02t0510200-01;v=rs18706774;vf=4199070)A[T](http://plants.ensembl.org/Oryza_sativa/ZMenu/TextSequence?db=core;factorytype=Location;g=Os02g0510200;r=2:18236073-18238350;t=Os02t0510200-01;v=rs18706783;vf=4199071)[G](http://plants.ensembl.org/Oryza_sativa/ZMenu/TextSequence?db=core;factorytype=Location;g=Os02g0510200;r=2:18236073-18238350;t=Os02t0510200-01;v=rs18706791;vf=4199072)[T](http://plants.ensembl.org/Oryza_sativa/ZMenu/TextSequence?db=core;factorytype=Location;g=Os02g0510200;r=2:18236073-18238350;t=Os02t0510200-01;v=rs18706800;vf=4199073)[T](http://plants.ensembl.org/Oryza_sativa/ZMenu/TextSequence?db=core;factorytype=Location;g=Os02g0510200;r=2:18236073-18238350;t=Os02t0510200-01;v=rs18706809;vf=4199074)AA[G](http://plants.ensembl.org/Oryza_sativa/ZMenu/TextSequence?db=core;factorytype=Location;g=Os02g0510200;r=2:18236073-18238350;t=Os02t0510200-01;v=rs18706818;vf=4199075)[C](http://plants.ensembl.org/Oryza_sativa/ZMenu/TextSequence?db=core;factorytype=Location;g=Os02g0510200;r=2:18236073-18238350;t=Os02t0510200-01;v=rs18706827;vf=4199076)T[T](http://plants.ensembl.org/Oryza_sativa/ZMenu/TextSequence?db=core;factorytype=Location;g=Os02g0510200;r=2:18236073-18238350;t=Os02t0510200-01;v=rs18706836;vf=4199077)[G](http://plants.ensembl.org/Oryza_sativa/ZMenu/TextSequence?db=core;factorytype=Location;g=Os02g0510200;r=2:18236073-18238350;t=Os02t0510200-01;v=rs18706845;vf=4199078)C[T](http://plants.ensembl.org/Oryza_sativa/ZMenu/TextSequence?db=core;factorytype=Location;g=Os02g0510200;r=2:18236073-18238350;t=Os02t0510200-01;v=rs18706854;vf=4199079)TTACAGGGCT[T](http://plants.ensembl.org/Oryza_sativa/ZMenu/TextSequence?db=core;factorytype=Location;g=Os02g0510200;r=2:18236073-18238350;t=Os02t0510200-01;v=rs18706863;vf=4199080)GAATG[C](http://plants.ensembl.org/Oryza_sativa/ZMenu/TextSequence?db=core;factorytype=Location;g=Os02g0510200;r=2:18236073-18238350;t=Os02t0510200-01;v=rs18706872;vf=4199081)[T](http://plants.ensembl.org/Oryza_sativa/ZMenu/TextSequence?db=core;factorytype=Location;g=Os02g0510200;r=2:18236073-18238350;t=Os02t0510200-01;v=rs18706881;vf=4199082)CTGC[T](http://plants.ensembl.org/Oryza_sativa/ZMenu/TextSequence?db=core;factorytype=Location;g=Os02g0510200;r=2:18236073-18238350;t=Os02t0510200-01;v=rs18706890;vf=4199083)A[C](http://plants.ensembl.org/Oryza_sativa/ZMenu/TextSequence?db=core;factorytype=Location;g=Os02g0510200;r=2:18236073-18238350;t=Os02t0510200-01;v=rs18706899;vf=4199084)A[A](http://plants.ensembl.org/Oryza_sativa/ZMenu/TextSequence?db=core;factorytype=Location;g=Os02g0510200;r=2:18236073-18238350;t=Os02t0510200-01;v=rs18706908;vf=4199085)[C](http://plants.ensembl.org/Oryza_sativa/ZMenu/TextSequence?db=core;factorytype=Location;g=Os02g0510200;r=2:18236073-18238350;t=Os02t0510200-01;v=rs18706917;vf=4199086)[A](http://plants.ensembl.org/Oryza_sativa/ZMenu/TextSequence?db=core;factorytype=Location;g=Os02g0510200;r=2:18236073-18238350;t=Os02t0510200-01;v=rs18706926;vf=4199087)GAG[C](http://plants.ensembl.org/Oryza_sativa/ZMenu/TextSequence?db=core;factorytype=Location;g=Os02g0510200;r=2:18236073-18238350;t=Os02t0510200-01;v=rs18706935;vf=4199088)ACAACA[A](http://plants.ensembl.org/Oryza_sativa/ZMenu/TextSequence?db=core;factorytype=Location;g=Os02g0510200;r=2:18236073-18238350;t=Os02t0510200-01;v=rs18706944;vf=4199089)[A](http://plants.ensembl.org/Oryza_sativa/ZMenu/TextSequence?db=core;factorytype=Location;g=Os02g0510200;r=2:18236073-18238350;t=Os02t0510200-01;v=rs18706953;vf=4199090)GAC[A](http://plants.ensembl.org/Oryza_sativa/ZMenu/TextSequence?db=core;factorytype=Location;g=Os02g0510200;r=2:18236073-18238350;t=Os02t0510200-01;v=rs18706962;vf=4199091)[A](http://plants.ensembl.org/Oryza_sativa/ZMenu/TextSequence?db=core;factorytype=Location;g=Os02g0510200;r=2:18236073-18238350;t=Os02t0510200-01;v=rs18706971;vf=4199092)GTTC[T](http://plants.ensembl.org/Oryza_sativa/ZMenu/TextSequence?db=core;factorytype=Location;g=Os02g0510200;r=2:18236073-18238350;t=Os02t0510200-01;v=rs18706980;vf=4199093)GA[T](http://plants.ensembl.org/Oryza_sativa/ZMenu/TextSequence?db=core;factorytype=Location;g=Os02g0510200;r=2:18236073-18238350;t=Os02t0510200-01;v=rs18706989;vf=4199094)[T](http://plants.ensembl.org/Oryza_sativa/ZMenu/TextSequence?db=core;factorytype=Location;g=Os02g0510200;r=2:18236073-18238350;t=Os02t0510200-01;v=rs18706998;vf=4199095)[T](http://plants.ensembl.org/Oryza_sativa/ZMenu/TextSequence?db=core;factorytype=Location;g=Os02g0510200;r=2:18236073-18238350;t=Os02t0510200-01;v=rs18707007;vf=4199096)[T](http://plants.ensembl.org/Oryza_sativa/ZMenu/TextSequence?db=core;factorytype=Location;g=Os02g0510200;r=2:18236073-18238350;t=Os02t0510200-01;v=rs18707016;vf=4199097)AGTG[C](http://plants.ensembl.org/Oryza_sativa/ZMenu/TextSequence?db=core;factorytype=Location;g=Os02g0510200;r=2:18236073-18238350;t=Os02t0510200-01;v=rs18707025;vf=4199098)[A](http://plants.ensembl.org/Oryza_sativa/ZMenu/TextSequence?db=core;factorytype=Location;g=Os02g0510200;r=2:18236073-18238350;t=Os02t0510200-01;v=vcZ269LAY;vf=4199099)[T](http://plants.ensembl.org/Oryza_sativa/ZMenu/TextSequence?db=core;factorytype=Location;g=Os02g0510200;r=2:18236073-18238350;t=Os02t0510200-01;v=rs18707034;vf=4199100)GGCACA[A](http://plants.ensembl.org/Oryza_sativa/ZMenu/TextSequence?db=core;factorytype=Location;g=Os02g0510200;r=2:18236073-18238350;t=Os02t0510200-01;v=rs53623627;vf=4199101)TGAGTT[G](http://plants.ensembl.org/Oryza_sativa/ZMenu/TextSequence?db=core;factorytype=Location;g=Os02g0510200;r=2:18236073-18238350;t=Os02t0510200-01;v=rs18707043;vf=4199102)[G](http://plants.ensembl.org/Oryza_sativa/ZMenu/TextSequence?db=core;factorytype=Location;g=Os02g0510200;r=2:18236073-18238350;t=Os02t0510200-01;v=rs18707052;vf=4199103)ACC[A](http://plants.ensembl.org/Oryza_sativa/ZMenu/TextSequence?db=core;factorytype=Location;g=Os02g0510200;r=2:18236073-18238350;t=Os02t0510200-01;v=rs18707061;vf=4199104)[G](http://plants.ensembl.org/Oryza_sativa/ZMenu/TextSequence?db=core;factorytype=Location;g=Os02g0510200;r=2:18236073-18238350;t=Os02t0510200-01;v=rs18707070;vf=4199105)CAG[A](http://plants.ensembl.org/Oryza_sativa/ZMenu/TextSequence?db=core;factorytype=Location;g=Os02g0510200;r=2:18236073-18238350;t=Os02t0510200-01;v=rs18707079;vf=4199106)[A](http://plants.ensembl.org/Oryza_sativa/ZMenu/TextSequence?db=core;factorytype=Location;g=Os02g0510200;r=2:18236073-18238350;t=Os02t0510200-01;v=rs18707088;vf=4199107)GA[G](http://plants.ensembl.org/Oryza_sativa/ZMenu/TextSequence?db=core;factorytype=Location;g=Os02g0510200;r=2:18236073-18238350;t=Os02t0510200-01;v=rs18707097;vf=4199108)GGAGT[T](http://plants.ensembl.org/Oryza_sativa/ZMenu/TextSequence?db=core;factorytype=Location;g=Os02g0510200;r=2:18236073-18238350;t=Os02t0510200-01;v=rs18707106;vf=4199109)[T](http://plants.ensembl.org/Oryza_sativa/ZMenu/TextSequence?db=core;factorytype=Location;g=Os02g0510200;r=2:18236073-18238350;t=Os02t0510200-01;v=rs18707115;vf=4199110)C

P L G Y K T F G E E I P P Q Y A I Q V L D E L T K G E A I I A T G V G Q H Q M

[C](http://plants.ensembl.org/Oryza_sativa/ZMenu/TextSequence?db=core;factorytype=Location;g=Os02g0510200;r=2:18236073-18238350;t=Os02t0510200-01;v=rs18707124;vf=4199111)TCT[G](http://plants.ensembl.org/Oryza_sativa/ZMenu/TextSequence?db=core;factorytype=Location;g=Os02g0510200;r=2:18236073-18238350;t=Os02t0510200-01;v=rs18707133;vf=4199112)[G](http://plants.ensembl.org/Oryza_sativa/ZMenu/TextSequence?db=core;factorytype=Location;g=Os02g0510200;r=2:18236073-18238350;t=Os02t0510200-01;v=rs18707142;vf=4199113)GGTAC[A](http://plants.ensembl.org/Oryza_sativa/ZMenu/TextSequence?db=core;factorytype=Location;g=Os02g0510200;r=2:18236073-18238350;t=Os02t0510200-01;v=rs18707151;vf=4199114)[A](http://plants.ensembl.org/Oryza_sativa/ZMenu/TextSequence?db=core;factorytype=Location;g=Os02g0510200;r=2:18236073-18238350;t=Os02t0510200-01;v=rs18707160;vf=4199115)[A](http://plants.ensembl.org/Oryza_sativa/ZMenu/TextSequence?db=core;factorytype=Location;g=Os02g0510200;r=2:18236073-18238350;t=Os02t0510200-01;v=rs53565787;vf=4199116)[A](http://plants.ensembl.org/Oryza_sativa/ZMenu/TextSequence?db=core;factorytype=Location;g=Os02g0510200;r=2:18236073-18238350;t=Os02t0510200-01;v=rs18707169;vf=4199117)CT[T](http://plants.ensembl.org/Oryza_sativa/ZMenu/TextSequence?db=core;factorytype=Location;g=Os02g0510200;r=2:18236073-18238350;t=Os02t0510200-01;v=rs18707178;vf=4199118)[T](http://plants.ensembl.org/Oryza_sativa/ZMenu/TextSequence?db=core;factorytype=Location;g=Os02g0510200;r=2:18236073-18238350;t=Os02t0510200-01;v=rs18707187;vf=4199119)[T](http://plants.ensembl.org/Oryza_sativa/ZMenu/TextSequence?db=core;factorytype=Location;g=Os02g0510200;r=2:18236073-18238350;t=Os02t0510200-01;v=rs18707196;vf=4199120)[G](http://plants.ensembl.org/Oryza_sativa/ZMenu/TextSequence?db=core;factorytype=Location;g=Os02g0510200;r=2:18236073-18238350;t=Os02t0510200-01;v=rs18707205;vf=4199121)G[T](http://plants.ensembl.org/Oryza_sativa/ZMenu/TextSequence?db=core;factorytype=Location;g=Os02g0510200;r=2:18236073-18238350;t=Os02t0510200-01;v=rs18707214;vf=4199122)[G](http://plants.ensembl.org/Oryza_sativa/ZMenu/TextSequence?db=core;factorytype=Location;g=Os02g0510200;r=2:18236073-18238350;t=Os02t0510200-01;v=rs18707223;vf=4199123)A[A](http://plants.ensembl.org/Oryza_sativa/ZMenu/TextSequence?db=core;factorytype=Location;g=Os02g0510200;r=2:18236073-18238350;t=Os02t0510200-01;v=rs18707232;vf=4199124)G[A](http://plants.ensembl.org/Oryza_sativa/ZMenu/TextSequence?db=core;factorytype=Location;g=Os02g0510200;r=2:18236073-18238350;t=Os02t0510200-01;v=rs18707241;vf=4199125)GAT[C](http://plants.ensembl.org/Oryza_sativa/ZMenu/TextSequence?db=core;factorytype=Location;g=Os02g0510200;r=2:18236073-18238350;t=Os02t0510200-01;v=rs18707250;vf=4199126)[C](http://plants.ensembl.org/Oryza_sativa/ZMenu/TextSequence?db=core;factorytype=Location;g=Os02g0510200;r=2:18236073-18238350;t=Os02t0510200-01;v=rs18707259;vf=4199127)[C](http://plants.ensembl.org/Oryza_sativa/ZMenu/TextSequence?db=core;factorytype=Location;g=Os02g0510200;r=2:18236073-18238350;t=Os02t0510200-01;v=rs18707268;vf=4199128)[A](http://plants.ensembl.org/Oryza_sativa/ZMenu/TextSequence?db=core;factorytype=Location;g=Os02g0510200;r=2:18236073-18238350;t=Os02t0510200-01;v=rs18707277;vf=4199129)[C](http://plants.ensembl.org/Oryza_sativa/ZMenu/TextSequence?db=core;factorytype=Location;g=Os02g0510200;r=2:18236073-18238350;t=Os02t0510200-01;v=rs18707286;vf=4199130)C[G](http://plants.ensembl.org/Oryza_sativa/ZMenu/TextSequence?db=core;factorytype=Location;g=Os02g0510200;r=2:18236073-18238350;t=Os02t0510200-01;v=rs53463516;vf=4199131)C[A](http://plants.ensembl.org/Oryza_sativa/ZMenu/TextSequence?db=core;factorytype=Location;g=Os02g0510200;r=2:18236073-18238350;t=Os02t0510200-01;v=rs18707295;vf=4199132)[A](http://plants.ensembl.org/Oryza_sativa/ZMenu/TextSequence?db=core;factorytype=Location;g=Os02g0510200;r=2:18236073-18238350;t=Os02t0510200-01;v=rs18707304;vf=4199133)[T](http://plants.ensembl.org/Oryza_sativa/ZMenu/TextSequence?db=core;factorytype=Location;g=Os02g0510200;r=2:18236073-18238350;t=Os02t0510200-01;v=rs18707313;vf=4199134)[A](http://plants.ensembl.org/Oryza_sativa/ZMenu/TextSequence?db=core;factorytype=Location;g=Os02g0510200;r=2:18236073-18238350;t=Os02t0510200-01;v=rs18707322;vf=4199135)TGC[C](http://plants.ensembl.org/Oryza_sativa/ZMenu/TextSequence?db=core;factorytype=Location;g=Os02g0510200;r=2:18236073-18238350;t=Os02t0510200-01;v=rs52949624;vf=4199136)A[T](http://plants.ensembl.org/Oryza_sativa/ZMenu/TextSequence?db=core;factorytype=Location;g=Os02g0510200;r=2:18236073-18238350;t=Os02t0510200-01;v=rs18707331;vf=4199137)TC[A](http://plants.ensembl.org/Oryza_sativa/ZMenu/TextSequence?db=core;factorytype=Location;g=Os02g0510200;r=2:18236073-18238350;t=Os02t0510200-01;v=rs18707340;vf=4199138)GGTGCT[G](http://plants.ensembl.org/Oryza_sativa/ZMenu/TextSequence?db=core;factorytype=Location;g=Os02g0510200;r=2:18236073-18238350;t=Os02t0510200-01;v=rs18707349;vf=4199139)[G](http://plants.ensembl.org/Oryza_sativa/ZMenu/TextSequence?db=core;factorytype=Location;g=Os02g0510200;r=2:18236073-18238350;t=Os02t0510200-01;v=rs18707358;vf=4199140)ATG[A](http://plants.ensembl.org/Oryza_sativa/ZMenu/TextSequence?db=core;factorytype=Location;g=Os02g0510200;r=2:18236073-18238350;t=Os02t0510200-01;v=rs18707367;vf=4199141)[G](http://plants.ensembl.org/Oryza_sativa/ZMenu/TextSequence?db=core;factorytype=Location;g=Os02g0510200;r=2:18236073-18238350;t=Os02t0510200-01;v=rs54023838;vf=4199142)CT[G](http://plants.ensembl.org/Oryza_sativa/ZMenu/TextSequence?db=core;factorytype=Location;g=Os02g0510200;r=2:18236073-18238350;t=Os02t0510200-01;v=rs18707376;vf=4199143)AC[G](http://plants.ensembl.org/Oryza_sativa/ZMenu/TextSequence?db=core;factorytype=Location;g=Os02g0510200;r=2:18236073-18238350;t=Os02t0510200-01;v=rs18707385;vf=4199144)[A](http://plants.ensembl.org/Oryza_sativa/ZMenu/TextSequence?db=core;factorytype=Location;g=Os02g0510200;r=2:18236073-18238350;t=Os02t0510200-01;v=rs18707394;vf=4199145)AAGG[T](http://plants.ensembl.org/Oryza_sativa/ZMenu/TextSequence?db=core;factorytype=Location;g=Os02g0510200;r=2:18236073-18238350;t=Os02t0510200-01;v=rs18707403;vf=4199146)G[A](http://plants.ensembl.org/Oryza_sativa/ZMenu/TextSequence?db=core;factorytype=Location;g=Os02g0510200;r=2:18236073-18238350;t=Os02t0510200-01;v=rs18707412;vf=4199147)[G](http://plants.ensembl.org/Oryza_sativa/ZMenu/TextSequence?db=core;factorytype=Location;g=Os02g0510200;r=2:18236073-18238350;t=Os02t0510200-01;v=rs18707421;vf=4199148)[G](http://plants.ensembl.org/Oryza_sativa/ZMenu/TextSequence?db=core;factorytype=Location;g=Os02g0510200;r=2:18236073-18238350;t=Os02t0510200-01;v=rs18707430;vf=4199149)CA[A](http://plants.ensembl.org/Oryza_sativa/ZMenu/TextSequence?db=core;factorytype=Location;g=Os02g0510200;r=2:18236073-18238350;t=Os02t0510200-01;v=rs18707439;vf=4199150)T[C](http://plants.ensembl.org/Oryza_sativa/ZMenu/TextSequence?db=core;factorytype=Location;g=Os02g0510200;r=2:18236073-18238350;t=Os02t0510200-01;v=rs18707448;vf=4199151)[A](http://plants.ensembl.org/Oryza_sativa/ZMenu/TextSequence?db=core;factorytype=Location;g=Os02g0510200;r=2:18236073-18238350;t=Os02t0510200-01;v=rs18707457;vf=4199152)T[C](http://plants.ensembl.org/Oryza_sativa/ZMenu/TextSequence?db=core;factorytype=Location;g=Os02g0510200;r=2:18236073-18238350;t=Os02t0510200-01;v=rs18707466;vf=4199153)G[C](http://plants.ensembl.org/Oryza_sativa/ZMenu/TextSequence?db=core;factorytype=Location;g=Os02g0510200;r=2:18236073-18238350;t=Os02t0510200-01;v=rs18707475;vf=4199154)TA[C](http://plants.ensembl.org/Oryza_sativa/ZMenu/TextSequence?db=core;factorytype=Location;g=Os02g0510200;r=2:18236073-18238350;t=Os02t0510200-01;v=rs18707484;vf=4199155)TG[G](http://plants.ensembl.org/Oryza_sativa/ZMenu/TextSequence?db=core;factorytype=Location;g=Os02g0510200;r=2:18236073-18238350;t=Os02t0510200-01;v=rs18707493;vf=4199156)TG[T](http://plants.ensembl.org/Oryza_sativa/ZMenu/TextSequence?db=core;factorytype=Location;g=Os02g0510200;r=2:18236073-18238350;t=Os02t0510200-01;v=rs18707502;vf=4199157)[T](http://plants.ensembl.org/Oryza_sativa/ZMenu/TextSequence?db=core;factorytype=Location;g=Os02g0510200;r=2:18236073-18238350;t=Os02t0510200-01;v=rs18707511;vf=4199158)GG[G](http://plants.ensembl.org/Oryza_sativa/ZMenu/TextSequence?db=core;factorytype=Location;g=Os02g0510200;r=2:18236073-18238350;t=Os02t0510200-01;v=rs18707520;vf=4199159)[C](http://plants.ensembl.org/Oryza_sativa/ZMenu/TextSequence?db=core;factorytype=Location;g=Os02g0510200;r=2:18236073-18238350;t=Os02t0510200-01;v=rs18707529;vf=4199160)AGC[A](http://plants.ensembl.org/Oryza_sativa/ZMenu/TextSequence?db=core;factorytype=Location;g=Os02g0510200;r=2:18236073-18238350;t=Os02t0510200-01;v=rs18707538;vf=4199161)CC[A](http://plants.ensembl.org/Oryza_sativa/ZMenu/TextSequence?db=core;factorytype=Location;g=Os02g0510200;r=2:18236073-18238350;t=Os02t0510200-01;v=rs18707547;vf=4199162)[G](http://plants.ensembl.org/Oryza_sativa/ZMenu/TextSequence?db=core;factorytype=Location;g=Os02g0510200;r=2:18236073-18238350;t=Os02t0510200-01;v=rs18707556;vf=4199163)ATG

W A A Q Y Y T Y K R P R Q W L S S A G L G A M G F G L P A A A G A S V A N P G

[T](http://plants.ensembl.org/Oryza_sativa/ZMenu/TextSequence?db=core;factorytype=Location;g=Os02g0510200;r=2:18236073-18238350;t=Os02t0510200-01;v=rs18707565;vf=4199164)[G](http://plants.ensembl.org/Oryza_sativa/ZMenu/TextSequence?db=core;factorytype=Location;g=Os02g0510200;r=2:18236073-18238350;t=Os02t0510200-01;v=rs18707573;vf=4199165)[G](http://plants.ensembl.org/Oryza_sativa/ZMenu/TextSequence?db=core;factorytype=Location;g=Os02g0510200;r=2:18236073-18238350;t=Os02t0510200-01;v=rs18707582;vf=4199166)[G](http://plants.ensembl.org/Oryza_sativa/ZMenu/TextSequence?db=core;factorytype=Location;g=Os02g0510200;r=2:18236073-18238350;t=Os02t0510200-01;v=rs18707591;vf=4199167)[C](http://plants.ensembl.org/Oryza_sativa/ZMenu/TextSequence?db=core;factorytype=Location;g=Os02g0510200;r=2:18236073-18238350;t=Os02t0510200-01;v=rs18707600;vf=4199168)[G](http://plants.ensembl.org/Oryza_sativa/ZMenu/TextSequence?db=core;factorytype=Location;g=Os02g0510200;r=2:18236073-18238350;t=Os02t0510200-01;v=rs18707609;vf=4199169)GCA[C](http://plants.ensembl.org/Oryza_sativa/ZMenu/TextSequence?db=core;factorytype=Location;g=Os02g0510200;r=2:18236073-18238350;t=Os02t0510200-01;v=rs18707620;vf=4199170)[A](http://plants.ensembl.org/Oryza_sativa/ZMenu/TextSequence?db=core;factorytype=Location;g=Os02g0510200;r=2:18236073-18238350;t=Os02t0510200-01;v=rs18707629;vf=4199171)A[T](http://plants.ensembl.org/Oryza_sativa/ZMenu/TextSequence?db=core;factorytype=Location;g=Os02g0510200;r=2:18236073-18238350;t=Os02t0510200-01;v=rs18707638;vf=4199172)[A](http://plants.ensembl.org/Oryza_sativa/ZMenu/TextSequence?db=core;factorytype=Location;g=Os02g0510200;r=2:18236073-18238350;t=Os02t0510200-01;v=rs18707647;vf=4199173)T[T](http://plants.ensembl.org/Oryza_sativa/ZMenu/TextSequence?db=core;factorytype=Location;g=Os02g0510200;r=2:18236073-18238350;t=Os02t0510200-01;v=rs18707656;vf=4199174)[A](http://plants.ensembl.org/Oryza_sativa/ZMenu/TextSequence?db=core;factorytype=Location;g=Os02g0510200;r=2:18236073-18238350;t=Os02t0510200-01;v=rs18707665;vf=4199175)CA[C](http://plants.ensembl.org/Oryza_sativa/ZMenu/TextSequence?db=core;factorytype=Location;g=Os02g0510200;r=2:18236073-18238350;t=Os02t0510200-01;v=rs18707674;vf=4199176)[C](http://plants.ensembl.org/Oryza_sativa/ZMenu/TextSequence?db=core;factorytype=Location;g=Os02g0510200;r=2:18236073-18238350;t=Os02t0510200-01;v=rs18707683;vf=4199177)[T](http://plants.ensembl.org/Oryza_sativa/ZMenu/TextSequence?db=core;factorytype=Location;g=Os02g0510200;r=2:18236073-18238350;t=Os02t0510200-01;v=rs18707692;vf=4199178)A[C](http://plants.ensembl.org/Oryza_sativa/ZMenu/TextSequence?db=core;factorytype=Location;g=Os02g0510200;r=2:18236073-18238350;t=Os02t0510200-01;v=rs18707701;vf=4199179)[A](http://plants.ensembl.org/Oryza_sativa/ZMenu/TextSequence?db=core;factorytype=Location;g=Os02g0510200;r=2:18236073-18238350;t=Os02t0510200-01;v=rs18707710;vf=4199180)[A](http://plants.ensembl.org/Oryza_sativa/ZMenu/TextSequence?db=core;factorytype=Location;g=Os02g0510200;r=2:18236073-18238350;t=Os02t0510200-01;v=rs18707719;vf=4199181)GC[G](http://plants.ensembl.org/Oryza_sativa/ZMenu/TextSequence?db=core;factorytype=Location;g=Os02g0510200;r=2:18236073-18238350;t=Os02t0510200-01;v=rs18707728;vf=4199182)[G](http://plants.ensembl.org/Oryza_sativa/ZMenu/TextSequence?db=core;factorytype=Location;g=Os02g0510200;r=2:18236073-18238350;t=Os02t0510200-01;v=rs18707737;vf=4199183)[C](http://plants.ensembl.org/Oryza_sativa/ZMenu/TextSequence?db=core;factorytype=Location;g=Os02g0510200;r=2:18236073-18238350;t=Os02t0510200-01;v=rs18707746;vf=4199184)[C](http://plants.ensembl.org/Oryza_sativa/ZMenu/TextSequence?db=core;factorytype=Location;g=Os02g0510200;r=2:18236073-18238350;t=Os02t0510200-01;v=rs18707755;vf=4199185)A[C](http://plants.ensembl.org/Oryza_sativa/ZMenu/TextSequence?db=core;factorytype=Location;g=Os02g0510200;r=2:18236073-18238350;t=Os02t0510200-01;v=rs18707764;vf=4199186)[G](http://plants.ensembl.org/Oryza_sativa/ZMenu/TextSequence?db=core;factorytype=Location;g=Os02g0510200;r=2:18236073-18238350;t=Os02t0510200-01;v=rs18707773;vf=4199187)[G](http://plants.ensembl.org/Oryza_sativa/ZMenu/TextSequence?db=core;factorytype=Location;g=Os02g0510200;r=2:18236073-18238350;t=Os02t0510200-01;v=rs18707782;vf=4199188)C[A](http://plants.ensembl.org/Oryza_sativa/ZMenu/TextSequence?db=core;factorytype=Location;g=Os02g0510200;r=2:18236073-18238350;t=Os02t0510200-01;v=rs18707791;vf=4199189)GT[G](http://plants.ensembl.org/Oryza_sativa/ZMenu/TextSequence?db=core;factorytype=Location;g=Os02g0510200;r=2:18236073-18238350;t=Os02t0510200-01;v=rs18707800;vf=4199190)[G](http://plants.ensembl.org/Oryza_sativa/ZMenu/TextSequence?db=core;factorytype=Location;g=Os02g0510200;r=2:18236073-18238350;t=Os02t0510200-01;v=rs18707809;vf=4199191)CTGT[C](http://plants.ensembl.org/Oryza_sativa/ZMenu/TextSequence?db=core;factorytype=Location;g=Os02g0510200;r=2:18236073-18238350;t=Os02t0510200-01;v=rs18707818;vf=4199192)[T](http://plants.ensembl.org/Oryza_sativa/ZMenu/TextSequence?db=core;factorytype=Location;g=Os02g0510200;r=2:18236073-18238350;t=Os02t0510200-01;v=rs18707827;vf=4199193)[T](http://plants.ensembl.org/Oryza_sativa/ZMenu/TextSequence?db=core;factorytype=Location;g=Os02g0510200;r=2:18236073-18238350;t=Os02t0510200-01;v=rs18707836;vf=4199194)[C](http://plants.ensembl.org/Oryza_sativa/ZMenu/TextSequence?db=core;factorytype=Location;g=Os02g0510200;r=2:18236073-18238350;t=Os02t0510200-01;v=rs18707845;vf=4199195)GG[C](http://plants.ensembl.org/Oryza_sativa/ZMenu/TextSequence?db=core;factorytype=Location;g=Os02g0510200;r=2:18236073-18238350;t=Os02t0510200-01;v=rs18707854;vf=4199196)TGGT[C](http://plants.ensembl.org/Oryza_sativa/ZMenu/TextSequence?db=core;factorytype=Location;g=Os02g0510200;r=2:18236073-18238350;t=Os02t0510200-01;v=rs18707863;vf=4199197)T[G](http://plants.ensembl.org/Oryza_sativa/ZMenu/TextSequence?db=core;factorytype=Location;g=Os02g0510200;r=2:18236073-18238350;t=Os02t0510200-01;v=rs18707872;vf=4199198)G[G](http://plants.ensembl.org/Oryza_sativa/ZMenu/TextSequence?db=core;factorytype=Location;g=Os02g0510200;r=2:18236073-18238350;t=Os02t0510200-01;v=rs18707881;vf=4199199)[C](http://plants.ensembl.org/Oryza_sativa/ZMenu/TextSequence?db=core;factorytype=Location;g=Os02g0510200;r=2:18236073-18238350;t=Os02t0510200-01;v=rs18707890;vf=4199200)GCAA[T](http://plants.ensembl.org/Oryza_sativa/ZMenu/TextSequence?db=core;factorytype=Location;g=Os02g0510200;r=2:18236073-18238350;t=Os02t0510200-01;v=rs18707899;vf=4199201)[G](http://plants.ensembl.org/Oryza_sativa/ZMenu/TextSequence?db=core;factorytype=Location;g=Os02g0510200;r=2:18236073-18238350;t=Os02t0510200-01;v=rs18707908;vf=4199202)[G](http://plants.ensembl.org/Oryza_sativa/ZMenu/TextSequence?db=core;factorytype=Location;g=Os02g0510200;r=2:18236073-18238350;t=Os02t0510200-01;v=rs18707917;vf=4199203)[G](http://plants.ensembl.org/Oryza_sativa/ZMenu/TextSequence?db=core;factorytype=Location;g=Os02g0510200;r=2:18236073-18238350;t=Os02t0510200-01;v=rs18707926;vf=4199204)ATTTGGG[C](http://plants.ensembl.org/Oryza_sativa/ZMenu/TextSequence?db=core;factorytype=Location;g=Os02g0510200;r=2:18236073-18238350;t=Os02t0510200-01;v=rs18707935;vf=4199205)TG[C](http://plants.ensembl.org/Oryza_sativa/ZMenu/TextSequence?db=core;factorytype=Location;g=Os02g0510200;r=2:18236073-18238350;t=Os02t0510200-01;v=rs18707944;vf=4199206)CTG[C](http://plants.ensembl.org/Oryza_sativa/ZMenu/TextSequence?db=core;factorytype=Location;g=Os02g0510200;r=2:18236073-18238350;t=Os02t0510200-01;v=rs18707953;vf=4199207)TGCA[G](http://plants.ensembl.org/Oryza_sativa/ZMenu/TextSequence?db=core;factorytype=Location;g=Os02g0510200;r=2:18236073-18238350;t=Os02t0510200-01;v=rs18707962;vf=4199208)[C](http://plants.ensembl.org/Oryza_sativa/ZMenu/TextSequence?db=core;factorytype=Location;g=Os02g0510200;r=2:18236073-18238350;t=Os02t0510200-01;v=rs18707971;vf=4199209)[T](http://plants.ensembl.org/Oryza_sativa/ZMenu/TextSequence?db=core;factorytype=Location;g=Os02g0510200;r=2:18236073-18238350;t=Os02t0510200-01;v=rs18707980;vf=4199210)GG[T](http://plants.ensembl.org/Oryza_sativa/ZMenu/TextSequence?db=core;factorytype=Location;g=Os02g0510200;r=2:18236073-18238350;t=Os02t0510200-01;v=rs18707989;vf=4199211)G[C](http://plants.ensembl.org/Oryza_sativa/ZMenu/TextSequence?db=core;factorytype=Location;g=Os02g0510200;r=2:18236073-18238350;t=Os02t0510200-01;v=rs18707998;vf=4199212)[T](http://plants.ensembl.org/Oryza_sativa/ZMenu/TextSequence?db=core;factorytype=Location;g=Os02g0510200;r=2:18236073-18238350;t=Os02t0510200-01;v=rs18708007;vf=4199213)T[C](http://plants.ensembl.org/Oryza_sativa/ZMenu/TextSequence?db=core;factorytype=Location;g=Os02g0510200;r=2:18236073-18238350;t=Os02t0510200-01;v=rs18708016;vf=4199214)[T](http://plants.ensembl.org/Oryza_sativa/ZMenu/TextSequence?db=core;factorytype=Location;g=Os02g0510200;r=2:18236073-18238350;t=Os02t0510200-01;v=rs18708025;vf=4199215)GT[G](http://plants.ensembl.org/Oryza_sativa/ZMenu/TextSequence?db=core;factorytype=Location;g=Os02g0510200;r=2:18236073-18238350;t=Os02t0510200-01;v=rs18708034;vf=4199216)GCT[A](http://plants.ensembl.org/Oryza_sativa/ZMenu/TextSequence?db=core;factorytype=Location;g=Os02g0510200;r=2:18236073-18238350;t=Os02t0510200-01;v=rs18708043;vf=4199217)AC[C](http://plants.ensembl.org/Oryza_sativa/ZMenu/TextSequence?db=core;factorytype=Location;g=Os02g0510200;r=2:18236073-18238350;t=Os02t0510200-01;v=rs18708052;vf=4199218)CAGG

V T V V D I D G D G S F L M N I Q E L A L I R I E N L P V K V M V L N N Q H

[T](http://plants.ensembl.org/Oryza_sativa/ZMenu/TextSequence?db=core;factorytype=Location;g=Os02g0510200;r=2:18236073-18238350;t=Os02t0510200-01;v=rs18708061;vf=4199219)G[T](http://plants.ensembl.org/Oryza_sativa/ZMenu/TextSequence?db=core;factorytype=Location;g=Os02g0510200;r=2:18236073-18238350;t=Os02t0510200-01;v=rs18708070;vf=4199220)CA[C](http://plants.ensembl.org/Oryza_sativa/ZMenu/TextSequence?db=core;factorytype=Location;g=Os02g0510200;r=2:18236073-18238350;t=Os02t0510200-01;v=rs18708079;vf=4199221)AG[T](http://plants.ensembl.org/Oryza_sativa/ZMenu/TextSequence?db=core;factorytype=Location;g=Os02g0510200;r=2:18236073-18238350;t=Os02t0510200-01;v=rs18708088;vf=4199222)[T](http://plants.ensembl.org/Oryza_sativa/ZMenu/TextSequence?db=core;factorytype=Location;g=Os02g0510200;r=2:18236073-18238350;t=Os02t0510200-01;v=rs18708097;vf=4199223)G[T](http://plants.ensembl.org/Oryza_sativa/ZMenu/TextSequence?db=core;factorytype=Location;g=Os02g0510200;r=2:18236073-18238350;t=Os02t0510200-01;v=rs18708106;vf=4199224)[T](http://plants.ensembl.org/Oryza_sativa/ZMenu/TextSequence?db=core;factorytype=Location;g=Os02g0510200;r=2:18236073-18238350;t=Os02t0510200-01;v=rs18708115;vf=4199225)[G](http://plants.ensembl.org/Oryza_sativa/ZMenu/TextSequence?db=core;factorytype=Location;g=Os02g0510200;r=2:18236073-18238350;t=Os02t0510200-01;v=rs18708124;vf=4199226)A[T](http://plants.ensembl.org/Oryza_sativa/ZMenu/TextSequence?db=core;factorytype=Location;g=Os02g0510200;r=2:18236073-18238350;t=Os02t0510200-01;v=rs18708133;vf=4199227)AT[T](http://plants.ensembl.org/Oryza_sativa/ZMenu/TextSequence?db=core;factorytype=Location;g=Os02g0510200;r=2:18236073-18238350;t=Os02t0510200-01;v=rs18708142;vf=4199228)[G](http://plants.ensembl.org/Oryza_sativa/ZMenu/TextSequence?db=core;factorytype=Location;g=Os02g0510200;r=2:18236073-18238350;t=Os02t0510200-01;v=rs18708150;vf=4199229)[A](http://plants.ensembl.org/Oryza_sativa/ZMenu/TextSequence?db=core;factorytype=Location;g=Os02g0510200;r=2:18236073-18238350;t=Os02t0510200-01;v=rs18708159;vf=4199230)TGGG[G](http://plants.ensembl.org/Oryza_sativa/ZMenu/TextSequence?db=core;factorytype=Location;g=Os02g0510200;r=2:18236073-18238350;t=Os02t0510200-01;v=rs18708168;vf=4199231)A[T](http://plants.ensembl.org/Oryza_sativa/ZMenu/TextSequence?db=core;factorytype=Location;g=Os02g0510200;r=2:18236073-18238350;t=Os02t0510200-01;v=rs18708177;vf=4199232)GG[T](http://plants.ensembl.org/Oryza_sativa/ZMenu/TextSequence?db=core;factorytype=Location;g=Os02g0510200;r=2:18236073-18238350;t=Os02t0510200-01;v=rs18708186;vf=4199233)A[G](http://plants.ensembl.org/Oryza_sativa/ZMenu/TextSequence?db=core;factorytype=Location;g=Os02g0510200;r=2:18236073-18238350;t=Os02t0510200-01;v=rs18708195;vf=4199234)[C](http://plants.ensembl.org/Oryza_sativa/ZMenu/TextSequence?db=core;factorytype=Location;g=Os02g0510200;r=2:18236073-18238350;t=Os02t0510200-01;v=rs18708204;vf=4199235)T[T](http://plants.ensembl.org/Oryza_sativa/ZMenu/TextSequence?db=core;factorytype=Location;g=Os02g0510200;r=2:18236073-18238350;t=Os02t0510200-01;v=rs18708213;vf=4199236)[C](http://plants.ensembl.org/Oryza_sativa/ZMenu/TextSequence?db=core;factorytype=Location;g=Os02g0510200;r=2:18236073-18238350;t=Os02t0510200-01;v=rs18708222;vf=4199237)[C](http://plants.ensembl.org/Oryza_sativa/ZMenu/TextSequence?db=core;factorytype=Location;g=Os02g0510200;r=2:18236073-18238350;t=Os02t0510200-01;v=rs18708231;vf=4199238)[T](http://plants.ensembl.org/Oryza_sativa/ZMenu/TextSequence?db=core;factorytype=Location;g=Os02g0510200;r=2:18236073-18238350;t=Os02t0510200-01;v=rs18708240;vf=4199239)C[A](http://plants.ensembl.org/Oryza_sativa/ZMenu/TextSequence?db=core;factorytype=Location;g=Os02g0510200;r=2:18236073-18238350;t=Os02t0510200-01;v=rs18708249;vf=4199240)[T](http://plants.ensembl.org/Oryza_sativa/ZMenu/TextSequence?db=core;factorytype=Location;g=Os02g0510200;r=2:18236073-18238350;t=Os02t0510200-01;v=rs18708258;vf=4199241)GAA[C](http://plants.ensembl.org/Oryza_sativa/ZMenu/TextSequence?db=core;factorytype=Location;g=Os02g0510200;r=2:18236073-18238350;t=Os02t0510200-01;v=rs18708267;vf=4199242)AT[T](http://plants.ensembl.org/Oryza_sativa/ZMenu/TextSequence?db=core;factorytype=Location;g=Os02g0510200;r=2:18236073-18238350;t=Os02t0510200-01;v=rs18708276;vf=4199243)CAG[G](http://plants.ensembl.org/Oryza_sativa/ZMenu/TextSequence?db=core;factorytype=Location;g=Os02g0510200;r=2:18236073-18238350;t=Os02t0510200-01;v=rs18708285;vf=4199244)[A](http://plants.ensembl.org/Oryza_sativa/ZMenu/TextSequence?db=core;factorytype=Location;g=Os02g0510200;r=2:18236073-18238350;t=Os02t0510200-01;v=rs18708294;vf=4199245)[G](http://plants.ensembl.org/Oryza_sativa/ZMenu/TextSequence?db=core;factorytype=Location;g=Os02g0510200;r=2:18236073-18238350;t=Os02t0510200-01;v=rs18708303;vf=4199246)[C](http://plants.ensembl.org/Oryza_sativa/ZMenu/TextSequence?db=core;factorytype=Location;g=Os02g0510200;r=2:18236073-18238350;t=Os02t0510200-01;v=rs53420886;vf=4199247)TGGCATTG[A](http://plants.ensembl.org/Oryza_sativa/ZMenu/TextSequence?db=core;factorytype=Location;g=Os02g0510200;r=2:18236073-18238350;t=Os02t0510200-01;v=rs18708312;vf=4199248)T[C](http://plants.ensembl.org/Oryza_sativa/ZMenu/TextSequence?db=core;factorytype=Location;g=Os02g0510200;r=2:18236073-18238350;t=Os02t0510200-01;v=rs18708321;vf=4199249)[C](http://plants.ensembl.org/Oryza_sativa/ZMenu/TextSequence?db=core;factorytype=Location;g=Os02g0510200;r=2:18236073-18238350;t=Os02t0510200-01;v=rs18708330;vf=4199250)G[C](http://plants.ensembl.org/Oryza_sativa/ZMenu/TextSequence?db=core;factorytype=Location;g=Os02g0510200;r=2:18236073-18238350;t=Os02t0510200-01;v=rs18708339;vf=4199251)[A](http://plants.ensembl.org/Oryza_sativa/ZMenu/TextSequence?db=core;factorytype=Location;g=Os02g0510200;r=2:18236073-18238350;t=Os02t0510200-01;v=rs18708348;vf=4199252)[T](http://plants.ensembl.org/Oryza_sativa/ZMenu/TextSequence?db=core;factorytype=Location;g=Os02g0510200;r=2:18236073-18238350;t=Os02t0510200-01;v=rs18708357;vf=4199253)T[G](http://plants.ensembl.org/Oryza_sativa/ZMenu/TextSequence?db=core;factorytype=Location;g=Os02g0510200;r=2:18236073-18238350;t=Os02t0510200-01;v=rs18708366;vf=4199254)[A](http://plants.ensembl.org/Oryza_sativa/ZMenu/TextSequence?db=core;factorytype=Location;g=Os02g0510200;r=2:18236073-18238350;t=Os02t0510200-01;v=rs18708375;vf=4199255)GAA[C](http://plants.ensembl.org/Oryza_sativa/ZMenu/TextSequence?db=core;factorytype=Location;g=Os02g0510200;r=2:18236073-18238350;t=Os02t0510200-01;v=rs18708384;vf=4199256)[C](http://plants.ensembl.org/Oryza_sativa/ZMenu/TextSequence?db=core;factorytype=Location;g=Os02g0510200;r=2:18236073-18238350;t=Os02t0510200-01;v=rs18708393;vf=4199257)T[C](http://plants.ensembl.org/Oryza_sativa/ZMenu/TextSequence?db=core;factorytype=Location;g=Os02g0510200;r=2:18236073-18238350;t=Os02t0510200-01;v=rs18708402;vf=4199258)CC[T](http://plants.ensembl.org/Oryza_sativa/ZMenu/TextSequence?db=core;factorytype=Location;g=Os02g0510200;r=2:18236073-18238350;t=Os02t0510200-01;v=rs53673823;vf=4199259)GTG[A](http://plants.ensembl.org/Oryza_sativa/ZMenu/TextSequence?db=core;factorytype=Location;g=Os02g0510200;r=2:18236073-18238350;t=Os02t0510200-01;v=rs18708411;vf=4199260)AGGT[G](http://plants.ensembl.org/Oryza_sativa/ZMenu/TextSequence?db=core;factorytype=Location;g=Os02g0510200;r=2:18236073-18238350;t=Os02t0510200-01;v=rs18708420;vf=4199261)[A](http://plants.ensembl.org/Oryza_sativa/ZMenu/TextSequence?db=core;factorytype=Location;g=Os02g0510200;r=2:18236073-18238350;t=Os02t0510200-01;v=rs18708429;vf=4199262)TGGTGTTGAACAA[C](http://plants.ensembl.org/Oryza_sativa/ZMenu/TextSequence?db=core;factorytype=Location;g=Os02g0510200;r=2:18236073-18238350;t=Os02t0510200-01;v=rs18708438;vf=4199263)[C](http://plants.ensembl.org/Oryza_sativa/ZMenu/TextSequence?db=core;factorytype=Location;g=Os02g0510200;r=2:18236073-18238350;t=Os02t0510200-01;v=rs18708447;vf=4199264)AA[C](http://plants.ensembl.org/Oryza_sativa/ZMenu/TextSequence?db=core;factorytype=Location;g=Os02g0510200;r=2:18236073-18238350;t=Os02t0510200-01;v=rs18708456;vf=4199265)A[T](http://plants.ensembl.org/Oryza_sativa/ZMenu/TextSequence?db=core;factorytype=Location;g=Os02g0510200;r=2:18236073-18238350;t=Os02t0510200-01;v=rs18708465;vf=4199266)T

L G M V V Q **W** E D R F Y K A N R A H T Y L G N P E C E S E I Y P D F V T I A K

[T](http://plants.ensembl.org/Oryza_sativa/ZMenu/TextSequence?db=core;factorytype=Location;g=Os02g0510200;r=2:18236073-18238350;t=Os02t0510200-01;v=rs18708474;vf=4199267)[G](http://plants.ensembl.org/Oryza_sativa/ZMenu/TextSequence?db=core;factorytype=Location;g=Os02g0510200;r=2:18236073-18238350;t=Os02t0510200-01;v=rs18708483;vf=4199268)[G](http://plants.ensembl.org/Oryza_sativa/ZMenu/TextSequence?db=core;factorytype=Location;g=Os02g0510200;r=2:18236073-18238350;t=Os02t0510200-01;v=rs18708492;vf=4199269)GT[A](http://plants.ensembl.org/Oryza_sativa/ZMenu/TextSequence?db=core;factorytype=Location;g=Os02g0510200;r=2:18236073-18238350;t=Os02t0510200-01;v=rs18708501;vf=4199270)[T](http://plants.ensembl.org/Oryza_sativa/ZMenu/TextSequence?db=core;factorytype=Location;g=Os02g0510200;r=2:18236073-18238350;t=Os02t0510200-01;v=rs18708510;vf=4199271)GG[T](http://plants.ensembl.org/Oryza_sativa/ZMenu/TextSequence?db=core;factorytype=Location;g=Os02g0510200;r=2:18236073-18238350;t=Os02t0510200-01;v=rs18708519;vf=4199272)[G](http://plants.ensembl.org/Oryza_sativa/ZMenu/TextSequence?db=core;factorytype=Location;g=Os02g0510200;r=2:18236073-18238350;t=Os02t0510200-01;v=rs18708528;vf=4199273)G[T](http://plants.ensembl.org/Oryza_sativa/ZMenu/TextSequence?db=core;factorytype=Location;g=Os02g0510200;r=2:18236073-18238350;t=Os02t0510200-01;v=rs18708537;vf=4199274)[G](http://plants.ensembl.org/Oryza_sativa/ZMenu/TextSequence?db=core;factorytype=Location;g=Os02g0510200;r=2:18236073-18238350;t=Os02t0510200-01;v=rs18708546;vf=4199275)CAA[**T**](http://plants.ensembl.org/Oryza_sativa/ZMenu/TextSequence?db=core;factorytype=Location;g=Os02g0510200;r=2:18236073-18238350;t=Os02t0510200-01;v=rs18708555;vf=4199276)[**G**](http://plants.ensembl.org/Oryza_sativa/ZMenu/TextSequence?db=core;factorytype=Location;g=Os02g0510200;r=2:18236073-18238350;t=Os02t0510200-01;v=rs18708564;vf=4199277)[**G**](http://plants.ensembl.org/Oryza_sativa/ZMenu/TextSequence?db=core;factorytype=Location;g=Os02g0510200;r=2:18236073-18238350;t=Os02t0510200-01;v=rs18708573;vf=4199278)GA[G](http://plants.ensembl.org/Oryza_sativa/ZMenu/TextSequence?db=core;factorytype=Location;g=Os02g0510200;r=2:18236073-18238350;t=Os02t0510200-01;v=rs18708582;vf=4199279)G[A](http://plants.ensembl.org/Oryza_sativa/ZMenu/TextSequence?db=core;factorytype=Location;g=Os02g0510200;r=2:18236073-18238350;t=Os02t0510200-01;v=rs18708591;vf=4199280)T[A](http://plants.ensembl.org/Oryza_sativa/ZMenu/TextSequence?db=core;factorytype=Location;g=Os02g0510200;r=2:18236073-18238350;t=Os02t0510200-01;v=rs18708600;vf=4199281)G[G](http://plants.ensembl.org/Oryza_sativa/ZMenu/TextSequence?db=core;factorytype=Location;g=Os02g0510200;r=2:18236073-18238350;t=Os02t0510200-01;v=rs18708609;vf=4199282)[T](http://plants.ensembl.org/Oryza_sativa/ZMenu/TextSequence?db=core;factorytype=Location;g=Os02g0510200;r=2:18236073-18238350;t=Os02t0510200-01;v=rs18708618;vf=4199283)[T](http://plants.ensembl.org/Oryza_sativa/ZMenu/TextSequence?db=core;factorytype=Location;g=Os02g0510200;r=2:18236073-18238350;t=Os02t0510200-01;v=rs18708627;vf=4199284)[T](http://plants.ensembl.org/Oryza_sativa/ZMenu/TextSequence?db=core;factorytype=Location;g=Os02g0510200;r=2:18236073-18238350;t=Os02t0510200-01;v=rs18708636;vf=4199285)T[A](http://plants.ensembl.org/Oryza_sativa/ZMenu/TextSequence?db=core;factorytype=Location;g=Os02g0510200;r=2:18236073-18238350;t=Os02t0510200-01;v=rs18708645;vf=4199286)CA[A](http://plants.ensembl.org/Oryza_sativa/ZMenu/TextSequence?db=core;factorytype=Location;g=Os02g0510200;r=2:18236073-18238350;t=Os02t0510200-01;v=rs18708654;vf=4199287)GGC[G](http://plants.ensembl.org/Oryza_sativa/ZMenu/TextSequence?db=core;factorytype=Location;g=Os02g0510200;r=2:18236073-18238350;t=Os02t0510200-01;v=rs18708663;vf=4199288)AA[T](http://plants.ensembl.org/Oryza_sativa/ZMenu/TextSequence?db=core;factorytype=Location;g=Os02g0510200;r=2:18236073-18238350;t=Os02t0510200-01;v=rs18708672;vf=4199289)[A](http://plants.ensembl.org/Oryza_sativa/ZMenu/TextSequence?db=core;factorytype=Location;g=Os02g0510200;r=2:18236073-18238350;t=Os02t0510200-01;v=rs18708681;vf=4199290)[G](http://plants.ensembl.org/Oryza_sativa/ZMenu/TextSequence?db=core;factorytype=Location;g=Os02g0510200;r=2:18236073-18238350;t=Os02t0510200-01;v=rs18708690;vf=4199291)G[G](http://plants.ensembl.org/Oryza_sativa/ZMenu/TextSequence?db=core;factorytype=Location;g=Os02g0510200;r=2:18236073-18238350;t=Os02t0510200-01;v=rs18708699;vf=4199292)[C](http://plants.ensembl.org/Oryza_sativa/ZMenu/TextSequence?db=core;factorytype=Location;g=Os02g0510200;r=2:18236073-18238350;t=Os02t0510200-01;v=rs18708708;vf=4199293)[G](http://plants.ensembl.org/Oryza_sativa/ZMenu/TextSequence?db=core;factorytype=Location;g=Os02g0510200;r=2:18236073-18238350;t=Os02t0510200-01;v=rs53284568;vf=4199294)[C](http://plants.ensembl.org/Oryza_sativa/ZMenu/TextSequence?db=core;factorytype=Location;g=Os02g0510200;r=2:18236073-18238350;t=Os02t0510200-01;v=rs18708717;vf=4199295)ATACAT[A](http://plants.ensembl.org/Oryza_sativa/ZMenu/TextSequence?db=core;factorytype=Location;g=Os02g0510200;r=2:18236073-18238350;t=Os02t0510200-01;v=rs18708726;vf=4199296)[C](http://plants.ensembl.org/Oryza_sativa/ZMenu/TextSequence?db=core;factorytype=Location;g=Os02g0510200;r=2:18236073-18238350;t=Os02t0510200-01;v=rs18708735;vf=4199297)T[T](http://plants.ensembl.org/Oryza_sativa/ZMenu/TextSequence?db=core;factorytype=Location;g=Os02g0510200;r=2:18236073-18238350;t=Os02t0510200-01;v=rs18708744;vf=4199298)[G](http://plants.ensembl.org/Oryza_sativa/ZMenu/TextSequence?db=core;factorytype=Location;g=Os02g0510200;r=2:18236073-18238350;t=Os02t0510200-01;v=rs18708753;vf=4199299)[G](http://plants.ensembl.org/Oryza_sativa/ZMenu/TextSequence?db=core;factorytype=Location;g=Os02g0510200;r=2:18236073-18238350;t=Os02t0510200-01;v=rs18708762;vf=4199300)GCAA[C](http://plants.ensembl.org/Oryza_sativa/ZMenu/TextSequence?db=core;factorytype=Location;g=Os02g0510200;r=2:18236073-18238350;t=Os02t0510200-01;v=rs53260769;vf=4199301)C[C](http://plants.ensembl.org/Oryza_sativa/ZMenu/TextSequence?db=core;factorytype=Location;g=Os02g0510200;r=2:18236073-18238350;t=Os02t0510200-01;v=rs18708771;vf=4199302)[G](http://plants.ensembl.org/Oryza_sativa/ZMenu/TextSequence?db=core;factorytype=Location;g=Os02g0510200;r=2:18236073-18238350;t=Os02t0510200-01;v=rs18708780;vf=4199303)GA[A](http://plants.ensembl.org/Oryza_sativa/ZMenu/TextSequence?db=core;factorytype=Location;g=Os02g0510200;r=2:18236073-18238350;t=Os02t0510200-01;v=rs18708789;vf=4199304)[T](http://plants.ensembl.org/Oryza_sativa/ZMenu/TextSequence?db=core;factorytype=Location;g=Os02g0510200;r=2:18236073-18238350;t=Os02t0510200-01;v=rs18708798;vf=4199305)[G](http://plants.ensembl.org/Oryza_sativa/ZMenu/TextSequence?db=core;factorytype=Location;g=Os02g0510200;r=2:18236073-18238350;t=Os02t0510200-01;v=rs18708807;vf=4199306)TG[A](http://plants.ensembl.org/Oryza_sativa/ZMenu/TextSequence?db=core;factorytype=Location;g=Os02g0510200;r=2:18236073-18238350;t=Os02t0510200-01;v=rs18708816;vf=4199307)G[A](http://plants.ensembl.org/Oryza_sativa/ZMenu/TextSequence?db=core;factorytype=Location;g=Os02g0510200;r=2:18236073-18238350;t=Os02t0510200-01;v=rs18708825;vf=4199308)G[C](http://plants.ensembl.org/Oryza_sativa/ZMenu/TextSequence?db=core;factorytype=Location;g=Os02g0510200;r=2:18236073-18238350;t=Os02t0510200-01;v=vcZ269LB8;vf=4199309)G[A](http://plants.ensembl.org/Oryza_sativa/ZMenu/TextSequence?db=core;factorytype=Location;g=Os02g0510200;r=2:18236073-18238350;t=Os02t0510200-01;v=rs18708834;vf=4199310)[G](http://plants.ensembl.org/Oryza_sativa/ZMenu/TextSequence?db=core;factorytype=Location;g=Os02g0510200;r=2:18236073-18238350;t=Os02t0510200-01;v=rs18708843;vf=4199311)ATATATCC[A](http://plants.ensembl.org/Oryza_sativa/ZMenu/TextSequence?db=core;factorytype=Location;g=Os02g0510200;r=2:18236073-18238350;t=Os02t0510200-01;v=rs18708852;vf=4199312)[G](http://plants.ensembl.org/Oryza_sativa/ZMenu/TextSequence?db=core;factorytype=Location;g=Os02g0510200;r=2:18236073-18238350;t=Os02t0510200-01;v=rs18708861;vf=4199313)A[T](http://plants.ensembl.org/Oryza_sativa/ZMenu/TextSequence?db=core;factorytype=Location;g=Os02g0510200;r=2:18236073-18238350;t=Os02t0510200-01;v=rs18708870;vf=4199314)T[T](http://plants.ensembl.org/Oryza_sativa/ZMenu/TextSequence?db=core;factorytype=Location;g=Os02g0510200;r=2:18236073-18238350;t=Os02t0510200-01;v=rs18708879;vf=4199315)[T](http://plants.ensembl.org/Oryza_sativa/ZMenu/TextSequence?db=core;factorytype=Location;g=Os02g0510200;r=2:18236073-18238350;t=Os02t0510200-01;v=rs18708888;vf=4199316)G[T](http://plants.ensembl.org/Oryza_sativa/ZMenu/TextSequence?db=core;factorytype=Location;g=Os02g0510200;r=2:18236073-18238350;t=Os02t0510200-01;v=rs18708897;vf=4199317)G[A](http://plants.ensembl.org/Oryza_sativa/ZMenu/TextSequence?db=core;factorytype=Location;g=Os02g0510200;r=2:18236073-18238350;t=Os02t0510200-01;v=rs18708906;vf=4199318)[C](http://plants.ensembl.org/Oryza_sativa/ZMenu/TextSequence?db=core;factorytype=Location;g=Os02g0510200;r=2:18236073-18238350;t=Os02t0510200-01;v=rs18708915;vf=4199319)[T](http://plants.ensembl.org/Oryza_sativa/ZMenu/TextSequence?db=core;factorytype=Location;g=Os02g0510200;r=2:18236073-18238350;t=Os02t0510200-01;v=rs18708924;vf=4199320)ATTGCTAA[G](http://plants.ensembl.org/Oryza_sativa/ZMenu/TextSequence?db=core;factorytype=Location;g=Os02g0510200;r=2:18236073-18238350;t=Os02t0510200-01;v=rs54310330;vf=4199321)

G F N I P A V R V T K K S E V R A A I K K M L E T P G P Y L L D I I V P H Q E

GG[G](http://plants.ensembl.org/Oryza_sativa/ZMenu/TextSequence?db=core;factorytype=Location;g=Os02g0510200;r=2:18236073-18238350;t=Os02t0510200-01;v=rs18708933;vf=4199322)TTCA[A](http://plants.ensembl.org/Oryza_sativa/ZMenu/TextSequence?db=core;factorytype=Location;g=Os02g0510200;r=2:18236073-18238350;t=Os02t0510200-01;v=rs18708942;vf=4199323)[T](http://plants.ensembl.org/Oryza_sativa/ZMenu/TextSequence?db=core;factorytype=Location;g=Os02g0510200;r=2:18236073-18238350;t=Os02t0510200-01;v=rs18708951;vf=4199324)ATTCCTG[C](http://plants.ensembl.org/Oryza_sativa/ZMenu/TextSequence?db=core;factorytype=Location;g=Os02g0510200;r=2:18236073-18238350;t=Os02t0510200-01;v=rs18708959;vf=4199325)AGT[C](http://plants.ensembl.org/Oryza_sativa/ZMenu/TextSequence?db=core;factorytype=Location;g=Os02g0510200;r=2:18236073-18238350;t=Os02t0510200-01;v=rs18708968;vf=4199326)CGTGTA[A](http://plants.ensembl.org/Oryza_sativa/ZMenu/TextSequence?db=core;factorytype=Location;g=Os02g0510200;r=2:18236073-18238350;t=Os02t0510200-01;v=rs18708977;vf=4199327)[C](http://plants.ensembl.org/Oryza_sativa/ZMenu/TextSequence?db=core;factorytype=Location;g=Os02g0510200;r=2:18236073-18238350;t=Os02t0510200-01;v=rs18708986;vf=4199328)[A](http://plants.ensembl.org/Oryza_sativa/ZMenu/TextSequence?db=core;factorytype=Location;g=Os02g0510200;r=2:18236073-18238350;t=Os02t0510200-01;v=rs18708995;vf=4199329)[A](http://plants.ensembl.org/Oryza_sativa/ZMenu/TextSequence?db=core;factorytype=Location;g=Os02g0510200;r=2:18236073-18238350;t=Os02t0510200-01;v=rs18709004;vf=4199330)AG[A](http://plants.ensembl.org/Oryza_sativa/ZMenu/TextSequence?db=core;factorytype=Location;g=Os02g0510200;r=2:18236073-18238350;t=Os02t0510200-01;v=rs18709013;vf=4199331)A[G](http://plants.ensembl.org/Oryza_sativa/ZMenu/TextSequence?db=core;factorytype=Location;g=Os02g0510200;r=2:18236073-18238350;t=Os02t0510200-01;v=rs18709022;vf=4199332)[A](http://plants.ensembl.org/Oryza_sativa/ZMenu/TextSequence?db=core;factorytype=Location;g=Os02g0510200;r=2:18236073-18238350;t=Os02t0510200-01;v=rs18709031;vf=4199333)GTG[A](http://plants.ensembl.org/Oryza_sativa/ZMenu/TextSequence?db=core;factorytype=Location;g=Os02g0510200;r=2:18236073-18238350;t=Os02t0510200-01;v=rs18709040;vf=4199334)A[G](http://plants.ensembl.org/Oryza_sativa/ZMenu/TextSequence?db=core;factorytype=Location;g=Os02g0510200;r=2:18236073-18238350;t=Os02t0510200-01;v=rs18709049;vf=4199335)T[C](http://plants.ensembl.org/Oryza_sativa/ZMenu/TextSequence?db=core;factorytype=Location;g=Os02g0510200;r=2:18236073-18238350;t=Os02t0510200-01;v=rs18709058;vf=4199336)[C](http://plants.ensembl.org/Oryza_sativa/ZMenu/TextSequence?db=core;factorytype=Location;g=Os02g0510200;r=2:18236073-18238350;t=Os02t0510200-01;v=rs18709067;vf=4199337)GTGC[C](http://plants.ensembl.org/Oryza_sativa/ZMenu/TextSequence?db=core;factorytype=Location;g=Os02g0510200;r=2:18236073-18238350;t=Os02t0510200-01;v=rs18709076;vf=4199338)[G](http://plants.ensembl.org/Oryza_sativa/ZMenu/TextSequence?db=core;factorytype=Location;g=Os02g0510200;r=2:18236073-18238350;t=Os02t0510200-01;v=rs18709085;vf=4199339)[C](http://plants.ensembl.org/Oryza_sativa/ZMenu/TextSequence?db=core;factorytype=Location;g=Os02g0510200;r=2:18236073-18238350;t=Os02t0510200-01;v=rs18709094;vf=4199340)[C](http://plants.ensembl.org/Oryza_sativa/ZMenu/TextSequence?db=core;factorytype=Location;g=Os02g0510200;r=2:18236073-18238350;t=Os02t0510200-01;v=rs18709103;vf=4199341)ATC[A](http://plants.ensembl.org/Oryza_sativa/ZMenu/TextSequence?db=core;factorytype=Location;g=Os02g0510200;r=2:18236073-18238350;t=Os02t0510200-01;v=rs18709112;vf=4199342)[A](http://plants.ensembl.org/Oryza_sativa/ZMenu/TextSequence?db=core;factorytype=Location;g=Os02g0510200;r=2:18236073-18238350;t=Os02t0510200-01;v=rs18709121;vf=4199343)[G](http://plants.ensembl.org/Oryza_sativa/ZMenu/TextSequence?db=core;factorytype=Location;g=Os02g0510200;r=2:18236073-18238350;t=Os02t0510200-01;v=rs18709130;vf=4199344)A[A](http://plants.ensembl.org/Oryza_sativa/ZMenu/TextSequence?db=core;factorytype=Location;g=Os02g0510200;r=2:18236073-18238350;t=Os02t0510200-01;v=rs18709139;vf=4199345)G[A](http://plants.ensembl.org/Oryza_sativa/ZMenu/TextSequence?db=core;factorytype=Location;g=Os02g0510200;r=2:18236073-18238350;t=Os02t0510200-01;v=rs18709148;vf=4199346)TG[C](http://plants.ensembl.org/Oryza_sativa/ZMenu/TextSequence?db=core;factorytype=Location;g=Os02g0510200;r=2:18236073-18238350;t=Os02t0510200-01;v=rs18709157;vf=4199347)TC[G](http://plants.ensembl.org/Oryza_sativa/ZMenu/TextSequence?db=core;factorytype=Location;g=Os02g0510200;r=2:18236073-18238350;t=Os02t0510200-01;v=rs18709166;vf=4199348)A[G](http://plants.ensembl.org/Oryza_sativa/ZMenu/TextSequence?db=core;factorytype=Location;g=Os02g0510200;r=2:18236073-18238350;t=Os02t0510200-01;v=10218237931;vf=4199349)[A](http://plants.ensembl.org/Oryza_sativa/ZMenu/TextSequence?db=core;factorytype=Location;g=Os02g0510200;r=2:18236073-18238350;t=Os02t0510200-01;v=rs18709184;vf=4199350)C[T](http://plants.ensembl.org/Oryza_sativa/ZMenu/TextSequence?db=core;factorytype=Location;g=Os02g0510200;r=2:18236073-18238350;t=Os02t0510200-01;v=rs18709193;vf=4199351)CCAG[G](http://plants.ensembl.org/Oryza_sativa/ZMenu/TextSequence?db=core;factorytype=Location;g=Os02g0510200;r=2:18236073-18238350;t=Os02t0510200-01;v=rs18709202;vf=4199352)GCC[A](http://plants.ensembl.org/Oryza_sativa/ZMenu/TextSequence?db=core;factorytype=Location;g=Os02g0510200;r=2:18236073-18238350;t=Os02t0510200-01;v=rs18709211;vf=4199353)[T](http://plants.ensembl.org/Oryza_sativa/ZMenu/TextSequence?db=core;factorytype=Location;g=Os02g0510200;r=2:18236073-18238350;t=Os02t0510200-01;v=rs18709220;vf=4199354)[A](http://plants.ensembl.org/Oryza_sativa/ZMenu/TextSequence?db=core;factorytype=Location;g=Os02g0510200;r=2:18236073-18238350;t=Os02t0510200-01;v=rs18709229;vf=4199355)CTTGTT[G](http://plants.ensembl.org/Oryza_sativa/ZMenu/TextSequence?db=core;factorytype=Location;g=Os02g0510200;r=2:18236073-18238350;t=Os02t0510200-01;v=rs18709238;vf=4199356)GA[T](http://plants.ensembl.org/Oryza_sativa/ZMenu/TextSequence?db=core;factorytype=Location;g=Os02g0510200;r=2:18236073-18238350;t=Os02t0510200-01;v=rs18709247;vf=4199357)[A](http://plants.ensembl.org/Oryza_sativa/ZMenu/TextSequence?db=core;factorytype=Location;g=Os02g0510200;r=2:18236073-18238350;t=Os02t0510200-01;v=rs18709256;vf=4199358)T[C](http://plants.ensembl.org/Oryza_sativa/ZMenu/TextSequence?db=core;factorytype=Location;g=Os02g0510200;r=2:18236073-18238350;t=Os02t0510200-01;v=rs18709265;vf=4199359)A[T](http://plants.ensembl.org/Oryza_sativa/ZMenu/TextSequence?db=core;factorytype=Location;g=Os02g0510200;r=2:18236073-18238350;t=Os02t0510200-01;v=rs18709274;vf=4199360)[C](http://plants.ensembl.org/Oryza_sativa/ZMenu/TextSequence?db=core;factorytype=Location;g=Os02g0510200;r=2:18236073-18238350;t=Os02t0510200-01;v=rs18709283;vf=4199361)G[T](http://plants.ensembl.org/Oryza_sativa/ZMenu/TextSequence?db=core;factorytype=Location;g=Os02g0510200;r=2:18236073-18238350;t=Os02t0510200-01;v=rs18709292;vf=4199362)CCC[G](http://plants.ensembl.org/Oryza_sativa/ZMenu/TextSequence?db=core;factorytype=Location;g=Os02g0510200;r=2:18236073-18238350;t=Os02t0510200-01;v=rs53620255;vf=4199363)[C](http://plants.ensembl.org/Oryza_sativa/ZMenu/TextSequence?db=core;factorytype=Location;g=Os02g0510200;r=2:18236073-18238350;t=Os02t0510200-01;v=rs18709301;vf=4199364)ACCA[G](http://plants.ensembl.org/Oryza_sativa/ZMenu/TextSequence?db=core;factorytype=Location;g=Os02g0510200;r=2:18236073-18238350;t=Os02t0510200-01;v=rs18709310;vf=4199365)GA

H V L P M I P **S G** G A F K D M I L D G D G R T V Y *

GCATG[T](http://plants.ensembl.org/Oryza_sativa/ZMenu/TextSequence?db=core;factorytype=Location;g=Os02g0510200;r=2:18236073-18238350;t=Os02t0510200-01;v=rs18709319;vf=4199366)[G](http://plants.ensembl.org/Oryza_sativa/ZMenu/TextSequence?db=core;factorytype=Location;g=Os02g0510200;r=2:18236073-18238350;t=Os02t0510200-01;v=rs18709328;vf=4199367)[C](http://plants.ensembl.org/Oryza_sativa/ZMenu/TextSequence?db=core;factorytype=Location;g=Os02g0510200;r=2:18236073-18238350;t=Os02t0510200-01;v=rs18709337;vf=4199368)TGCCTATGAT[C](http://plants.ensembl.org/Oryza_sativa/ZMenu/TextSequence?db=core;factorytype=Location;g=Os02g0510200;r=2:18236073-18238350;t=Os02t0510200-01;v=rs18709346;vf=4199369)[C](http://plants.ensembl.org/Oryza_sativa/ZMenu/TextSequence?db=core;factorytype=Location;g=Os02g0510200;r=2:18236073-18238350;t=Os02t0510200-01;v=rs18709355;vf=4199370)[C](http://plants.ensembl.org/Oryza_sativa/ZMenu/TextSequence?db=core;factorytype=Location;g=Os02g0510200;r=2:18236073-18238350;t=Os02t0510200-01;v=rs18709364;vf=4199371)[A](http://plants.ensembl.org/Oryza_sativa/ZMenu/TextSequence?db=core;factorytype=Location;g=Os02g0510200;r=2:18236073-18238350;t=Os02t0510200-01;v=rs18709373;vf=4199372)**A**[**G**](http://plants.ensembl.org/Oryza_sativa/ZMenu/TextSequence?db=core;factorytype=Location;g=Os02g0510200;r=2:18236073-18238350;t=Os02t0510200-01;v=vcZ269LBD;vf=4199373)**T**[**G**](http://plants.ensembl.org/Oryza_sativa/ZMenu/TextSequence?db=core;factorytype=Location;g=Os02g0510200;r=2:18236073-18238350;t=Os02t0510200-01;v=rs18709382;vf=4199374)[**G**](http://plants.ensembl.org/Oryza_sativa/ZMenu/TextSequence?db=core;factorytype=Location;g=Os02g0510200;r=2:18236073-18238350;t=Os02t0510200-01;v=rs18709391;vf=4199375)**G**[G](http://plants.ensembl.org/Oryza_sativa/ZMenu/TextSequence?db=core;factorytype=Location;g=Os02g0510200;r=2:18236073-18238350;t=Os02t0510200-01;v=rs18709400;vf=4199376)GC[G](http://plants.ensembl.org/Oryza_sativa/ZMenu/TextSequence?db=core;factorytype=Location;g=Os02g0510200;r=2:18236073-18238350;t=Os02t0510200-01;v=rs18709409;vf=4199377)C[A](http://plants.ensembl.org/Oryza_sativa/ZMenu/TextSequence?db=core;factorytype=Location;g=Os02g0510200;r=2:18236073-18238350;t=Os02t0510200-01;v=rs18709418;vf=4199378)[T](http://plants.ensembl.org/Oryza_sativa/ZMenu/TextSequence?db=core;factorytype=Location;g=Os02g0510200;r=2:18236073-18238350;t=Os02t0510200-01;v=rs18709427;vf=4199379)TCAA[G](http://plants.ensembl.org/Oryza_sativa/ZMenu/TextSequence?db=core;factorytype=Location;g=Os02g0510200;r=2:18236073-18238350;t=Os02t0510200-01;v=rs18709436;vf=4199380)[G](http://plants.ensembl.org/Oryza_sativa/ZMenu/TextSequence?db=core;factorytype=Location;g=Os02g0510200;r=2:18236073-18238350;t=Os02t0510200-01;v=rs18709445;vf=4199381)ACA[T](http://plants.ensembl.org/Oryza_sativa/ZMenu/TextSequence?db=core;factorytype=Location;g=Os02g0510200;r=2:18236073-18238350;t=Os02t0510200-01;v=rs18709454;vf=4199382)G[A](http://plants.ensembl.org/Oryza_sativa/ZMenu/TextSequence?db=core;factorytype=Location;g=Os02g0510200;r=2:18236073-18238350;t=Os02t0510200-01;v=rs18709463;vf=4199383)T[C](http://plants.ensembl.org/Oryza_sativa/ZMenu/TextSequence?db=core;factorytype=Location;g=Os02g0510200;r=2:18236073-18238350;t=Os02t0510200-01;v=rs18709472;vf=4199384)CT[G](http://plants.ensembl.org/Oryza_sativa/ZMenu/TextSequence?db=core;factorytype=Location;g=Os02g0510200;r=2:18236073-18238350;t=Os02t0510200-01;v=rs18709481;vf=4199385)[G](http://plants.ensembl.org/Oryza_sativa/ZMenu/TextSequence?db=core;factorytype=Location;g=Os02g0510200;r=2:18236073-18238350;t=Os02t0510200-01;v=rs18709490;vf=4199386)A[T](http://plants.ensembl.org/Oryza_sativa/ZMenu/TextSequence?db=core;factorytype=Location;g=Os02g0510200;r=2:18236073-18238350;t=Os02t0510200-01;v=rs18709499;vf=4199387)[G](http://plants.ensembl.org/Oryza_sativa/ZMenu/TextSequence?db=core;factorytype=Location;g=Os02g0510200;r=2:18236073-18238350;t=Os02t0510200-01;v=rs18709507;vf=4199388)[G](http://plants.ensembl.org/Oryza_sativa/ZMenu/TextSequence?db=core;factorytype=Location;g=Os02g0510200;r=2:18236073-18238350;t=Os02t0510200-01;v=rs18709516;vf=4199389)TGA[T](http://plants.ensembl.org/Oryza_sativa/ZMenu/TextSequence?db=core;factorytype=Location;g=Os02g0510200;r=2:18236073-18238350;t=Os02t0510200-01;v=rs18709525;vf=4199390)[G](http://plants.ensembl.org/Oryza_sativa/ZMenu/TextSequence?db=core;factorytype=Location;g=Os02g0510200;r=2:18236073-18238350;t=Os02t0510200-01;v=rs18709534;vf=4199391)GCA[G](http://plants.ensembl.org/Oryza_sativa/ZMenu/TextSequence?db=core;factorytype=Location;g=Os02g0510200;r=2:18236073-18238350;t=Os02t0510200-01;v=rs18709543;vf=4199392)GACT[G](http://plants.ensembl.org/Oryza_sativa/ZMenu/TextSequence?db=core;factorytype=Location;g=Os02g0510200;r=2:18236073-18238350;t=Os02t0510200-01;v=vcZ269LBE;vf=4199393)[T](http://plants.ensembl.org/Oryza_sativa/ZMenu/TextSequence?db=core;factorytype=Location;g=Os02g0510200;r=2:18236073-18238350;t=Os02t0510200-01;v=rs18709552;vf=4199394)GT[A](http://plants.ensembl.org/Oryza_sativa/ZMenu/TextSequence?db=core;factorytype=Location;g=Os02g0510200;r=2:18236073-18238350;t=Os02t0510200-01;v=rs18709561;vf=4199395)T**T**[**A**](http://plants.ensembl.org/Oryza_sativa/ZMenu/TextSequence?db=core;factorytype=Location;g=Os02g0510200;r=2:18236073-18238350;t=Os02t0510200-01;v=rs18709570;vf=4199396)[**A**](http://plants.ensembl.org/Oryza_sativa/ZMenu/TextSequence?db=core;factorytype=Location;g=Os02g0510200;r=2:18236073-18238350;t=Os02t0510200-01;v=rs18709579;vf=4199397)

Translated sequence

Variants MissenseStop gained Stop lostSynonymous

Positions of mutations conferring herbicide resistance in weed plants (8 plus Gly121) are highlighted in bold (nucleotides and amino acids) and red (amino acids)

Number of point mutation positions in the rice ALS CDS:

Non-synonymous 505 (including Missense 485 plus Nonsense/Sense = Stop gained/lost 20), synonymous 180

Total 685 (/1935 bp = 35.4%), non-synonymous/synonymous 2.8

Variation data were taken from six different large scale rice studies available in Ensembl Genomes (release 39):

1. The 3000 Rice Genome Project (2014), an international effort to sequence the genomes of 3,024 rice varieties from 89 countries providing 365,710 variant loci (SNPs and InDels).
2. Whole genome sequencing of 104 elite rice cultivars (Duitama et al. 2015), described as "a comprehensive information resource for marker assisted selection" providing 25,769,548 variant loci.
3. Chip based analysis of 1,310 SNPs across 395 samples (Zhao et al. 2010), described as, "revealing the impact of domestication and breeding on the rice genome".
4. Chip based analysis of approximately 160k SNPs across 20 diversity rice accessions (OryzaSNP, McNally et al. 2009), described as, "revealing relationships among landraces and modern varieties of rice".
5. The Oryza Map Alignment Project (OMAP, Wing et al. 2005): approximately 1.6M variant loci detected by comparing BAC End Sequences from four rice varieties to Japonica. [dbSNP]
6. Adaptive loss-of-function in domesticated rice (Yu et al. 2005): A collection of approximately 3M variant loci from the comparison of the Indica (93-11) and Japonica (‘Nipponbare’) genomes. [dbSNP]

The following genetic markers were remapped to the IRGSP-1.0 assembly by industry collaborator KeyGene:

- 20,483 Quantitative Trait Locus (QTL): 19,435 from Gramene’s legacy QTLs database and 1,048 from the Q-Taro database
- 1,278 genetic markers (990 RFLPs and 288 SSRs) from Gramene’s legacy markers database

References

Duitama J, Silva A, Sanabria Y, Cruz DF, Quintero C, Ballen C, Lorieux M, Scheffler B, Farmer A, Torres E et al. 2015. Whole genome sequencing of elite rice cultivars as a comprehensive information resource for marker assisted selection. PLoS ONE. 10:e0124617.

McNally KL, Childs KL, Bohnert R, Davidson RM, Zhao K, Ulat VJ, Zeller G, Clark RM, Hoen DR, Bureau TE et al. 2009. Genomewide SNP variation reveals relationships among landraces and modern varieties of rice. Proc. Natl. Acad. Sci. USA. 106:12273-12278.

The 3,000 rice genomes project. 2014. Gigascience. 3:7.

Wing RA, Ammiraju J, Luo M, Kim H, Yu Y, Kudrna D et al. (2005) The *Oryza* Map Alignment Project: the golden path to unlocking the genetic potential of wild rice species. Plant Mol. Biol. 59:53-62.

Yu J, Wang J, Lin W, Li S, Li H, Zhou J, Ni P, Dong W, Hu S, Zeng C et al. 2005. The genomes of *Oryza sativa*: a history of duplications. PLoS Biol. 3:e38.

Zhao K, Wright M, Kimball J, Eizenga G, McClung A, Kovach M, Tyagi W, Ali ML, Tung CW, Reynolds A et al. 2010. Genomic diversity and introgression in *O. sativa* reveal the impact of domestication and breeding on the rice genome. PLoS ONE. 5:e10780.
