# Supplementary material for: Genetic Alterations That Do or Do Not Occur Naturally; Consequences for Genome Edited Organisms in the Context of Regulatory Oversight
Source: Front Bioeng Biotechnol. 2019 Jan 16;6:213. doi: 10.3389/fbioe.2018.00213 (PMC6343457; doi:10.3389/fbioe.2018.00213)
Supplement: Supplementary file 4 [file Table_4.docx]

**Appendix: The occurrence of SNPs in the gene coding for acetolactate synthase (ALS) in Arabidopsis, wheat and rice**

Spontaneous SNPs were screened for in the ALS gene (coding sequence, CDS) in the *Arabidopsis thaliana* pangenome as well as in hexaploid wheat and rice genome sequences maintained at the Ensembl Genomes portal (release 39). The Arabidopsis variation database contained the latest 1001 variation data set, covering more than 10 million variant loci across 1,135 natural inbred lines (The 1001 Genomes Consortium, 2016). Out of 100 SNPs located in the ALS gene, which cover 5% of the full-length coding sequence, the majority (76) were synonymous and only 24 (25%) SNPs were non-synonymous (Table S1).

In wheat, more limited data were available, primarily representing inter-homoeologous (A, B and D subgenomic) variation in the ALS gene. Similarly to Arabidopsis, 78 SNP sites (4% of the coding sequence) were identified, of which more than 88% (69 sites) were synonymous (Table S2).

In rice, data for several thousands of *Oryza sativa* accessions were obtained, including those of the 3,000 Rice Genomes Project (2014, Wang et al., 2018). In total, 685 SNPs (35% of the 1,935-bp coding sequence) were identified, and 505 SNPs resulted in non-synonymous (missense and non-sense) alterations, that is 2.8 times higher than the 180 synonymous SNPs (Table S3).

The raw data from Tables S1-S3 for the three plant species are summarized in Table S4 below. As expected for accessions subject to natural selection, a similar and relatively low SNP frequency (4-5%) was observed in Arabidopsis and wheat with a high predominance for synonymous SNPs that preserve the function of the ALS gene. There were only three overlaps between Arabidopsis and wheat SNPs, leaving a total of about 170 ALS SNPs in the studied material, which indicates that spontaneous SNPs already can cover a significant (close to 10%) portion of the coding sequence.

In contrast, rice genomes are characterized by a much higher overall SNP frequency (35%) in the ALS gene and a massive prevalence for non-synonymous mutations (including seven sites of herbicide resistance). The reason for this discrepancy is not clear, but it may indicate the effect of human intervention since the core collection of 3,000+ sequenced accessions contains breeding lines and old or modern cultivars from 89 countries all over the world.

**Table S4.** Summary of spontaneous SNPs in the ALS gene of three plant species

HR, no. of sites of potential herbicide resistance; N/A, not applicable; *ALS gene on chromosome 6DL

References

The 1001 Genomes Consortium (2016). 1,135 genomes reveal the global pattern of polymorphism in *Arabidopsis thaliana*. *Cell* 166, 481–491. doi: 10.1016/j.cell.2016.05.063

The 3,000 rice genome project (2014). The 3,000 rice genome project. *GigaScience* 3, 7. doi: 10.1186/2047-217X-3-7

Wang, W., Mauleon, R., Hu, Z., Chebotarov, D., Tai, S., Wu, Z., et al. (2018). Genomic variation in 3,010 diverse accessions of Asian cultivated rice. *Nature* 557, 43–49. doi: 10.1038/s41586-018-0063-9
